# Rapid selection of sulphadoxine-resistant *Plasmodium falciparum* and its effect on within-population genetic diversity in Papua New Guinea

**DOI:** 10.1038/s41598-018-23811-7

**Published:** 2018-04-03

**Authors:** Toshihiro Mita, Francis Hombhanje, Nobuyuki Takahashi, Makoto Sekihara, Masato Yamauchi, Takahiro Tsukahara, Akira Kaneko, Hiroyoshi Endo, Jun Ohashi

**Affiliations:** 1 Department of Tropical Medicine and Parasitology, Juntendo University School of Medicine, 2-1-1 Hongo, Bunkyo, Tokyo, 113-8421 Japan; 2 Centre for Health Research & Diagnostics, Divine Word University, Nabasa Road, P.O. Box 483, Madang, Papua New Guinea; 3 Department of International Affairs and Tropical Medicine, Tokyo Women’s Medical University, 8-1 Kawada-cho, Shinjuku-ku, Tokyo, 162-8666 Japan; 4 Department of Microbiology, Tumor and Cell Biology, Karolinska Institutet, SE-171 77 Stockholm, Sweden; 5 Department of Parasitology, Osaka City University Graduate School of Medicine, Asahi-cho 1-4-3, Abeno-ku, Osaka, 545-8585 Japan; 6 Department of Biological Sciences, Graduate School of Science, The University of Tokyo, 7-3-1 Hongo, Bunkyo-ku, Tokyo, 113-0033 Japan

## Abstract

The ability of the human malarial parasite *Plasmodium falciparum* to adapt to environmental changes depends considerably on its ability to maintain within-population genetic variation. Strong selection, consequent to widespread antimalarial drug usage, occasionally elicits a rapid expansion of drug-resistant isolates, which can act as founders. To investigate whether this phenomenon induces a loss of within-population genetic variation, we performed a population genetic analysis on 302 *P*. *falciparum* cases detected during two cross-sectional surveys in 2002/2003, just after the official introduction of sulphadoxine/pyrimethamine as a first-line treatment, and again in 2010/2011, in highly endemic areas in Papua New Guinea. We found that a single-origin sulphadoxine-resistant parasite isolate rapidly increased from 0% in 2002/2003 to 54% in 2010 and 84% in 2011. However, a considerable number of pairs exhibited random associations among 10 neutral microsatellite markers located in various chromosomes, suggesting that outcrossing effectively reduced non-random associations, albeit at a low average multiplicity of infection (1.35–1.52). Within-population genetic diversity was maintained throughout the study period. This indicates that the parasites maintained within-population variation, even after a clonal expansion of drug-resistant parasites. Outcrossing played a role in the preservation of within-population genetic diversity despite low levels of multiplicity of infection.

## Introduction

Genetic diversity, or the extent of genetic variation within a population, is one of the factors necessary for organisms to adapt to environmental changes^1^. Malaria is caused by the protozoan parasite *Plasmodium*, and 216 million cases were reported worldwide in 2015, responsible for an estimated 445,000 deaths^2^. In high transmission areas, populations of *P*. *falciparum*, the species of *Plasmodium* responsible for the most lethal form of malaria, generally exhibit high levels of genetic diversity, which has enabled this parasite species to develop survival mechanisms such as antimalarial drug resistance and immune evasion^3–6^. Genetic diversity can be lost in response to several population genetic and environmental factors. In particular, reductions in population-size can potentially reduce genetic variation, an extreme case of which is known as the bottleneck effect^7^. In malarial parasites, reductions in population size are often induced by significant environmental changes, such as effective malaria control programs which reduce the vector mosquito population as well as the number of malaria-infected individuals. One factor that potentially creates population bottlenecks is widespread antimalarial drug use^8^. This is because, while susceptible parasites succumb to the selective pressure imposed by antimalarial drug usage, a small number of parasites resistant to the drugs survive and expand in the parasite population. However, since random recombination theoretically occurs in malaria parasites, the effect of selection is expected to be limited to genetic regions flanking drug resistance genes^9,10^, and selection is therefore not expected to have a global effect on genetic diversity and the genome in general. However, in the Greater Mekong Subregion, where *P*. *falciparum* parasites resistant to artemisinin combination therapies have emerged^11–13^, rapid expansion of artemisinin-resistant parasite numbers resulted in distinct but apparently sympatric parasite subpopulations with extremely high levels of genetic differentiation between them^14,15^. This further emphasizes the importance of investigating if the expansion of single origin drug-resistant parasite isolates contributes to the reduction of within-population genetic diversity in different transmission settings.

Papua New Guinea has the highest burden of malaria outside of Africa^2^. Overall, malaria transmission intensity is high, with limited seasonal fluctuations in the costal and lowland zones^5,16,17^. Malaria epidemiology is, however, substantially heterogeneous because of diverse ecological factors related to the malarial parasites themselves, mosquito vectors, human hosts, and the natural environment^16,18^. All four *Plasmodium* species that cause malaria in humans (*P*. *falciparum*, *P*. *vivax*, *P*. *ovale*, and *P*. *malariae*) are observed in this region^18^. An intense malaria control program was launched with the first country-wide free distribution of long-lasting insecticidal nets (LLIN) in 2004^19^, which led to substantial reduction in mosquito biting rates and entomological inoculation rates^20^ as well as in malaria incidence rates and malaria prevalence^21^. A combination of sulphadoxine/pyrimethamine along with one of the 4-aminoquinolines (chloroquine or amodiaquine) was used as a first-line treatment for uncomplicated malaria between 2000 to 2010. We previously conducted a study on the molecular epidemiology of drug resistance in the Wewak district of East Sepik in Papua New Guinea. Our study found that single-origin sulphadoxine-resistant *P*. *falciparum* parasites appeared in 2003, soon after the penetration of this treatment to the studied area^22^. However, it is not clear whether this rapid expansion of the sulphadoxine-resistant parasite isolate has affected local parasite populations and, in particular, whether it has contributed to the emergence of a subpopulation and caused reductions in within-population genetic variation. To assess this, we performed a population genetic analysis using *P*. *falciparum* isolates obtained from four cross-sectional surveys in the Boikin-Dagua Rural Local-Level Government area, East Sepik, which is 56 km away from the Wewak district (where the previous study was conducted in 2002 and 2003^22^) before and just after the rapid increase in sulphadoxine-resistant parasites.

## Results

### Prevalence of *P*. *falciparum* and multiplicity of infections (MOIs)

**Figure 1:**
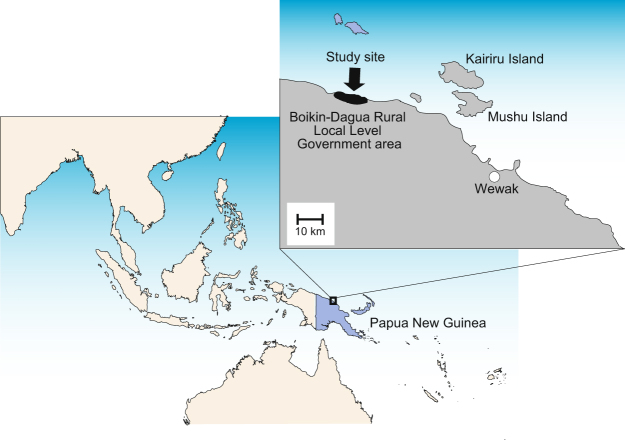
Location of the study site. Four cross-sectional surveys were performed at one catchment area including 14 villages (black colour) in the Boikin-Dagua Rural Local-Level Government area, East Sepik in Papua New Guinea. The map was created using Adobe Illustrator CC 19.2.1 (64-bit) (http://www.adobe.com/jp/products/illustrator.html).

The studies were conducted in a small catchment area spanning 15 km along the coast and containing 14 villages in the Boikin-Dagua Rural Local-Level Government area, East Sepik (Fig. 1). We recruited 2805 individuals without age restriction. Almost all recruited individuals were asymptomatic. Microscopic screening revealed that 22.4% of the individuals were malaria positive (*P*. *falciparum* 10.8%, *P*. *vivax* 8.8%, and *P*. *malariae* 2.8%) in 2002 and 20.1% (*P*. *falciparum* 13.1%, *P*. *vivax* 6.1%, and *P*. *malariae* 1.0%) in 2003 (Table 1). Microscopic examination was not performed in 2010 and did not produce malaria prevalence data of sufficient quality to use in 2011. We performed species-specific PCRs in 947 samples in which dried-blood samples were available, which confirmed 302 *P*. *falciparum* infection; 31 in 2002, 55 in 2003, 144 in 2010, and 72 in 2011 (Table 1). The prevalence of *P*. *falciparum* infections in 2002 (47.7%) and 2003 (42.3%) were significantly higher than those in 2010 (28.0%) and 2011 (30.3%) (2002/2003 vs 2010/2011, *P* < 0.001, Chi-square test).

**Table 1:** Characteristics of studied subjects.

	2002	2003	2010	2011
No. of recruied individuals	992	719	560	534
*Pf* prevalence by microscope (%)	10.8%	13.1%	ND	ND
*Pf* prevalence by PCR (%)	47.7%	42.3%	28.0%	30.3%
No. of *Pf* analyzed samples	31	55	144	72
Gender ratio (Female, %)	55%	60%	55%	56%
Avarage age (Year)	8.6	10.5	ND	6.7
Multiple-clonal infections (%)	45.2%	47.2%	38.9%	29.2%
MOI	1.52	1.64	1.41	1.35

ND: Not determined.

MOI: Multiplicity of infection.

MOIs were determined from ten neutral microsatellite markers by counting the greatest numbers of alleles at the most polymorphic locus (Table 1, Supplementary Fig. S1). This method provides the minimum number of clones per isolate. The maximum MOI detected was three and was observed in 18 samples. The prevalence of multiple-clonal infections (MOI > 1) significantly decreased from 2002 (45.2%) to 2011 (29.2%) (*P* = 0.04092, Cochran-Armitage trend test). Mean MOI was 1.52 in 2002, 1.64 in 2003, 1.41 in 2010, 1.35 in 2011 and a significant downward trend in mean MOIs (*P* = 0.009, Jonckheere-Terpstra trend test) was observed. We consider that distribution of LLIN as a part of National Malaria Control Programme launched in 2004, between our sampling times, had a significant impact on the observed reduction in prevalence of *P*. *falciparum* and MOI in 2010/2011.

### Allele prevalence and frequencies in ***pfcrt, dhfr***, and ***dhps***

**Figure 2:**
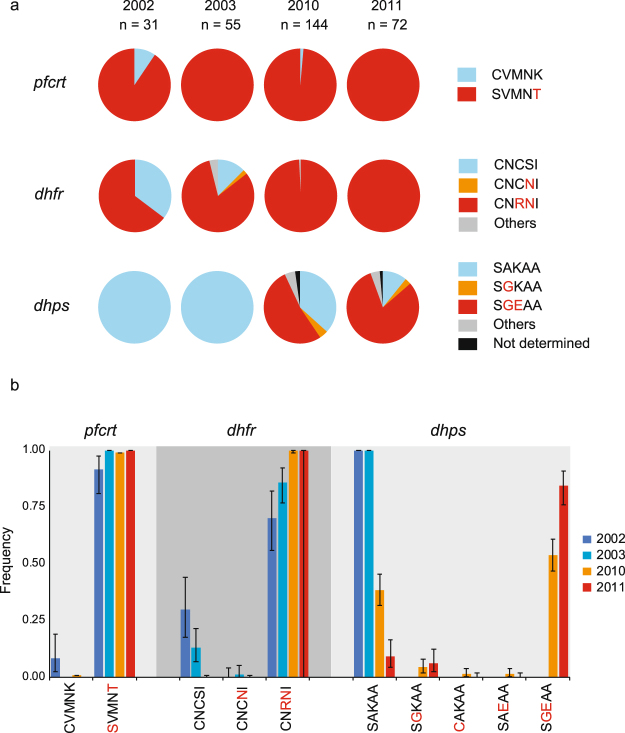
Changes in *pfcrt*, *dhfr*, and *dhps* allele prevalence and frequency in *Plasmodium falciparum* isolates. Capital letters denote the amino acid residues at positions 72–76 in *pfcrt*; 50, 51, 59, 108, and 164 in *dhfr*; and 436, 437, 540, 581, and 613 in *dhps;* mutations are identified in red. (**a**) Allele prevalence in *pfcrt*, *dhfr*, and *dhps*. Other denotations (grey colour) are the alleles of CNCSI + CNCNI (*n* = 2) in 2003 and CNCTI (*n* = 1) in 2010 in *dhfr* and CAKAA (*n* = 2), SAEAA (*n* = 1), and SAKAA + SAEAA (*n* = 1) in 2010 and SGKAA + SGEAA (*n* = 2) in *dhps*. (**b**) Allele frequencies in *pfcrt*, *dhfr*, and *dhps*, which were estimated by using a maximum likelihood algorithm in the MalHaploFreq program. Error bars represent the 95% confidence intervals for the estimated frequencies.

We estimated allele frequencies of *pfcrt*, *dhfr*, and *dhps* based on the prevalence of these alleles and MOIs using MalHaploFreq^23^ (Fig. 2). In *pfcrt*, the chloroquine-resistant SVMNT (amino acid positions 72–76; mutations underlined) was fixed or nearly fixed throughout the study period, probably owing to long-term chloroquine usage before our study. The sulphadoxine/pyrimethamine plus 4-aminoquinoline (chloroquine or amodiaquine) regimen was officially introduced as a first-line treatment for uncomplicated malaria in 2000, but the penetration of this change into the study area occurred sometime in 2002, concurrent with our sampling period. Before this change, sulphadoxine/pyrimethamine had been used infrequently as a second-line treatment with quinine. Notably, the increase in sulphadoxine/pyrimethamine selection pressure did not affect the frequencies of pyrimethamine- and sulphadoxine-resistant alleles equally. The frequency of the pyrimethamine-resistant *dhfr* allele (CNRNI: amino acids at positions 50, 51, 59, 108, and 164) was already high at the beginning of the study (70% in 2002 and 86% in 2003), and subsequently achieved fixation in 2010. In contrast, conspicuous changes in allele frequencies were observed with respect to sulphadoxine resistance. The sulphadoxine-resistant allele (SGEAA, amino acids at positions 436, 437, 540, 581, and 613) was not observed in 2002 and 2003. The frequency of this allele, however, reached 54% in 2010 and rapidly increased to 84% in 2011.

To investigate if this rapid increase in the frequency of the sulphadoxine-resistant allele was because of single-origin expansion, six microsatellite markers located near *dhps* were genotyped. A microsatellite haplotype (Common-type, 197-166-136-117-116-111 at −2.9 kb, −1.5 kb, −0.13 kb, +0.8 kb, +4.3 kb, and +7.7 kb, respectively) accounts for 87% of parasites harbouring the SGEAA mutation (Table 2). The remaining parasites (13%) belong to five variants of the Common-type. Among these variant-types, three (Variant-1, −2, and −3, n = 10) showed a 2-bp difference at polymorphic loci, suggesting that these differences were induced by slippage of microsatellites during meiosis. Of the other two parasites, one had alleles with 2-bp differences at −2.9 kb and −1.5 kb (Variant-4) and the other had a 10 bp-sized difference at 4.3 kb (Variant-5) from the Common-type. These results suggest that the SGEAA-type sulphadoxine-resistant allele was derived from a single origin in the studied population.

**Table 2:** Microsatellite haplotypes around *dhps* in *Plasmodium falciparum* isolates harboring an SGEAA allele.

	Microsatellite haplotypes							
	−2.9 kb	−1.5 kb	−0.13 kb	0.8 kb	4.3 kb	7.7 kb	*n* (2010)	*n* (2011)
Common-type	197	166	136	117	116	111	32	51
Variant-1	197	166	134	117	116	111	6	0
Variant-2	195	166	136	117	116	111	1	2
Variant-3	197	164	136	117	116	111	0	1
Variant-4	195	164	136	117	116	111	1	0
Variant-5	197	166	136	117	106	111	1	0

### Effect of rapid expansion of a sulphadoxine-resistant parasite isolate on the genetic diversity of parasite populations

**Figure 3:**
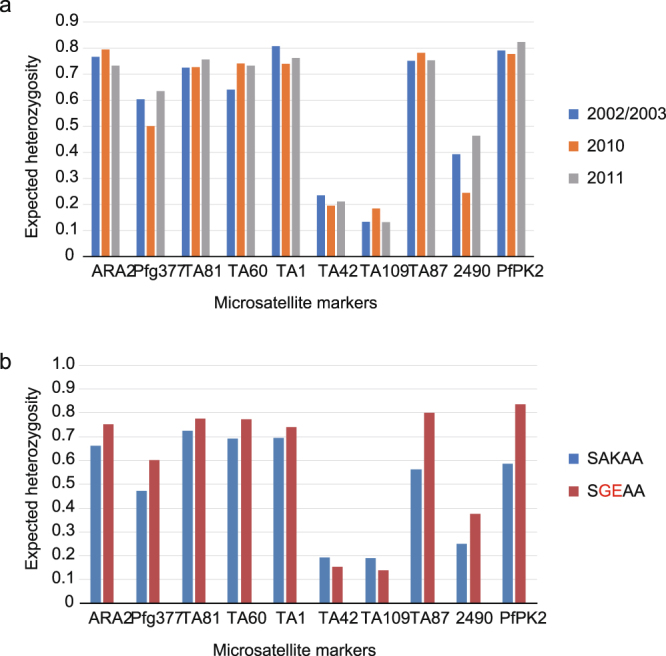
Expected heterozygosity of 10 microsatellite markers in *Plasmodium falciparum* isolates. (**a**) Isolates were classified into three groups; 2002/2003 (n = 86), 2010 (n = 144), and 2011 (n = 72). (**b**) Isolates were classified into two groups; wild-type (SAKAA, n = 62) and mutant (SGEAA, n = 136) in *dhps*.

To investigate if the rapid expansion of a single-origin sulphadoxine-resistant parasite isolate had induced a loss of within-population genetic diversity, we performed a population genetic analysis with 10 neutral microsatellites located on chromosomes other than that bearing *dhps*. We classified the samples into three groups according to the frequency of the sulphadoxine-resistant allele (SGEAA): 2002/2003 (0%), 2010 (54%), and 2011 (84%). In 2002/2003, high levels of expected heterozygosity (0.60–0.81) were observed at all but three loci (TA42, TA109, and 2490) (Fig. 3a). These high levels of expected heterozygosity were also observed in 2010 and 2011. Likewise, nearly all pairwise comparisons of expected heterozygosity at each microsatellite locus between the three groups (2002/2003 vs. 2010 vs. 2011) revealed non-statistically significant differences (Supplementary Fig. S2). This observation was supported by the values for the mean number of alleles per locus and allelic richness (number of alleles adjusted for sample size), which were also similar among the three groups (Table 3 and Supplementary Table S1). These results strongly suggest that the rapid expansion of a sulphadoxine-resistant parasite isolate did not cause a loss of within-population genetic variation on a genome-wide scale.

**Table 3:** Number of alleles, allele richness, effective population sizes and multilocus linkage disequilibrium assessments estimated from 10 neutral microsatellite markers.

	Years			***dhps*** ^a^	
	2002/2003	2010	2011	SAKAA	SGEAA
Average number of alleles/locus	7.2	7.2	7.1	6.1	8.1
Allele richness	7.0862	6.7074	7.089	6.1	7.25
Estimates of multilocus linkage disequilibrium					
Test factor					
V_D_	1.91	2.39	2.26	3.74	2.35
V_e_	1.87	1.76	1.80	2.08	1.75
I^A^_S_	0.0022	0.0394	0.0283	0.0886	0.0385
Testing (H_0_: V_D_ = V_e_)					
Var (V_D_)	0.01	6.5 × 10^−3^	1.0 × 10^−2^	6.5 × 10^−3^	6.5 × 10^−3^
P value	0.36	<1 × 10^−4^	1 × 10^−4^	<1 × 10^−4^	<1 × 10^−4^

^a^Analysis was carried out with the data from 2010 and 2011.

**Figure 4:**
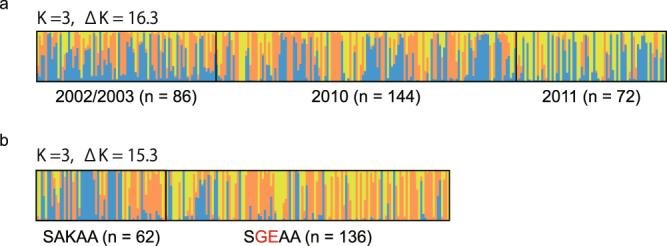
Bayesian population structure analysis of *Plasmodium falciparum* isolates. The highest value of ΔK occurred at K = 3 in both analysis by year (**a**) and by *dhps* genotype (wild-type and SGEAA mutant). Each individual is represented by a vertical bar displaying the proportion of membership to each of the clusters.

**Figure 5:**
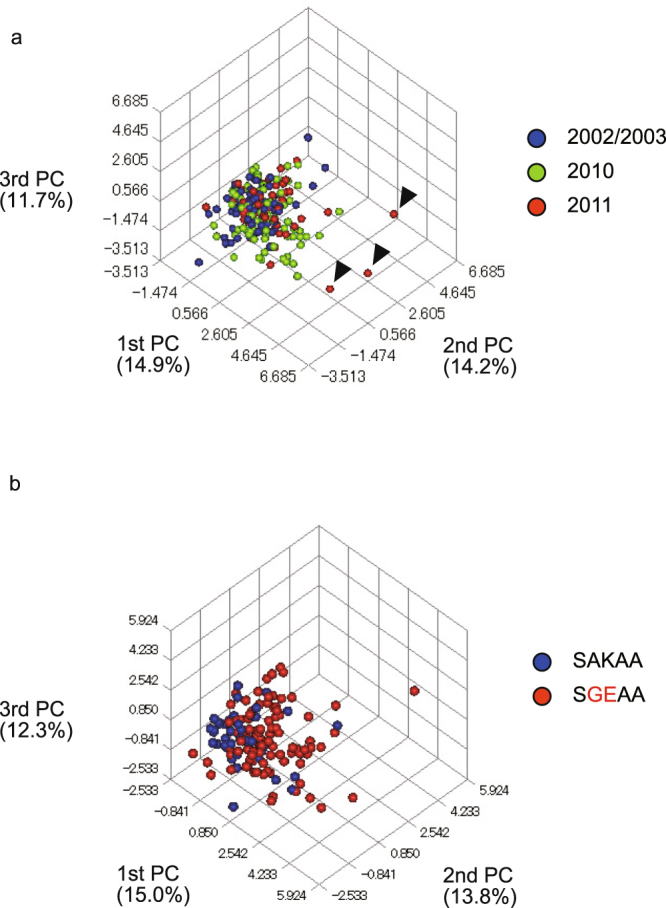
Principal component analysis of *Plasmodium falciparum* isolates. A scatter plot of the top three principal components from an analysis of 10 microsatellite markers with complete allele typing. Each point represents an isolate. Proportions of variance are described in parenthesis under principal component (PC). (**a**) A total of 263 isolates were included in the analysis (82 in 2002/2003, 120 in 2010 and 61 in 2011). (**b**) A total of 161 isolates harbouring wild-type (n = 50) or mutant SGEAA (n = 111) were included in the analysis. Since only wild-type was observed in 2002 and 2003, analysis was performed in 2010 and 2011.

There is a possibility that the observed preservation of high genetic variation in 2010 and 2011 is because of the mixture of distinct population(s) possessing high genetic variation. To assess this, we performed a widely used Bayesian clustering method, STRUCTURE analysis, which can detect a weak population structure. The result exhibited that the highest delta K was obtained at K = 3 in both the analysis with all isolates (Fig. 4a) and that with only the single-clonal isolates (Supplementary Fig. S3a). These clusters were observed in all three sample groups (2002/2003, 2010, and 2011). We also performed PCA (Principal component analysis) using 263 isolates without missing genotypes at 10 neutral microsatellite loci. The results again revealed no distinct clusters, regardless of their assigned group, in both the sample-set of all isolates (Fig. 5a) and single-clonal isolates (Supplementary Fig. S3b). All outliers (n = 3, Fig. 5a, arrow heads) also harboured a SGEAA allele with the Common microsatellite haplotype. These results exclude the possibility that the parasite populations in 2010 and 2011 were derived from a population distinct from that observed in 2002/2003.

Since the single-origin sulphadoxine-resistant parasites rapidly expanded in the studied area, a lower level of genetic variation was expected in the sulphadoxine-resistant parasite population than in the sulphadoxine-susceptible parasite population. However, high levels of expected heterozygosity (Fig. 3b) and allelic richness (Table 3) were found in the mutant (SGEAA) isolates and, notably, these values tend to be higher in *dhps* in the mutant isolates than in the wild-type isolates. This result was supported by PCA, which exhibited a more scattered distribution of mutant isolates than wild-type isolates (Fig. 5b). In the STRUCTURE analysis, three clusters were most probable, and all clusters were distributed in both the sulphadoxine-resistant and sulphadoxine-susceptible parasite populations (Fig. 4b).

### Effect of a rapid expansion of a sulphadoxine-resistant parasite isolate on the non-random association between neutral microsatellites in parasite populations

**Figure 6:**
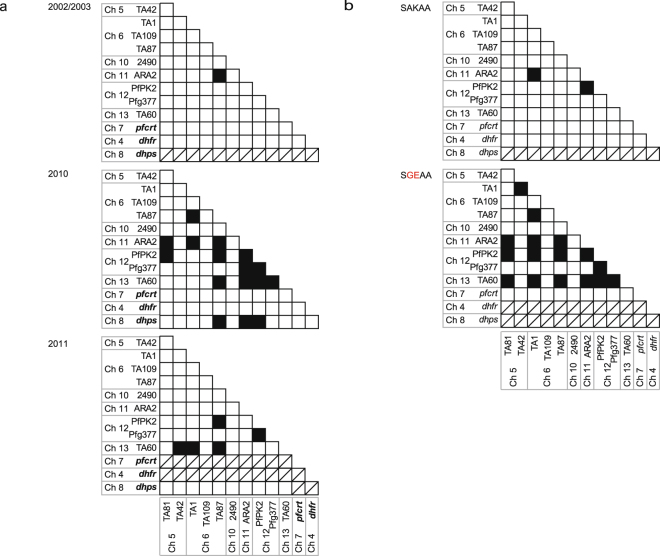
Association between pairs of 10 microsatellite markers, *pfcrt*, *dhfr*, and *dhps* in *Plasmodium falciparum* populations. (**a**) 2002/2003 (n = 86), 2010 (n = 144), and 2011 (n = 72) and (**b**) Two populations harbouring *dhps*- SGEAA (n = 136) and SAKAA (n = 62) were shown. Since the dhps-SGEAA mutant was not observed in 2002/2003, this analysis was carried out with the data from 2010 and 2011. Black colour denotes significant non-random associations. Bonferroni correction was applied to P values, with significance set at P < 0.00064 (2010), P < 0.000758 (2002/2003 and SAKAA), and P < 0.000943 (2011 and SGEAA). Oblique lines are the pairs in which non-random association was not calculated because of the absence of polymorphic locus (loci).

It is expected that a rapid expansion of sulphadoxine-resistant parasite isolates would induce non-random associations of unlinked loci when the prevalence of MOI is not high. To assess this, we first performed overall multilocus non-random association assessments using the locus of 10 neutral microsatellite markers, *pfcrt*, *dhfr*, and *dhps*. The results revealed no multilocus non-random association in 2002/2003 (*V*_*D*_ = 1.91, *P* < 0.36) but significant multilocus non-random association in 2010 (*V*_*D*_ = 2.39, *P* < 1 × 10^−4^) and 2011 (*V*_*D*_ = 2.26, *P* = 1 × 10^−4^) (Table 3, Supplementary Table S2). We next examined pairwise non-random associations between these loci. As expected, almost all locus pairs (65/66) exhibited random associations in 2002/2003. However, contrary to our expectation that few locus pairs would show random associations because of the rapid expansion of sulphadoxine-resistant parasites, as many as 79% (62/78) in 2010 and 91% (50/55) in 2011 showed random associations (Fig. 6a). Even when pairwise comparisons were limited to loci on the same chromosomes, random associations were detected in 60% (3/5) in 2010 and 80% (4/5) in 2011. In addition, pairs with non-random associations substantially decreased from 16 in 2010 to 5 in 2011 (Fig. 6a). Because malaria parasites perform sexual reproduction in the mosquito stage, we consider that the non-random associations between loci created by the rapid expansion of the single-origin sulphadoxine resistant isolates were disrupted by chromosome shuffling and recombination during non-self-fertilization.

This notion is supported by the comparison between parasites harbouring the *dhps*-SGEAA mutant and those harbouring the wild-type *dhps* allele. Despite the higher proportion of pairs which displayed random associations in the group with the wild-type *dhps* allele, as many as 29 pairs (64%) showed random associations in the parasite group with SGEAA (Fig. 6b).

## Discussion

Here we provide evidence that the introduction of sulphadoxine/pyrimethamine as a first-line treatment for malaria in Papua New Guinea has induced a rapid increase in *P*. *falciparum* isolates harbouring the sulphadoxine-resistant *dhps* mutation, and that all mutants share a common resistance origin in our study area. This rapid expansion of common-origin resistance in isolates evoked an increase in non-random associations between evolutionary-neutral, unlinked genetic markers. This effect, however, was not sufficient to reduce the levels of within-population genetic variation, suggesting robust maintenance of genetic variation on a genome-wide scale.

In our study area, sulphadoxine-resistant parasite isolates harbouring the *dhps* SGEAA allele rapidly increased from 0% in 2002/2003 to 54% in 2010 and 84% in 2011. These isolates had very similar haplotypes of microsatellite markers flanking *dhps*, which strongly suggests a single resistant origin, in agreement with a previous result obtained in the Wewak district^22^. In accordance with this, non-random associations were observed between some pairs of evolutionary-neutral microsatellite markers. It is well known that several evolutionary factors can cause the generation of non-random associations. These include multilocus selection of studied markers, population hybridization, and rapid expansion of a clonal population^7,24^. However, multilocus selection is a less feasible explanation for this phenomenon since all microsatellite markers analysed in this study were believed to be evolutionarily neutral^3,25^. A recent population hybridization was also negated by PCA and a Bayesian clustering method. A more plausible explanation of the non-random associations between neutral microsatellites is the rapid expansion of the clonal population.

In spite of this, the levels of within-population genetic variation were not reduced. We consider that multiple instances of outcrossing between drug-resistant and drug-susceptible parasite isolates might have occurred just after the emergence of the single-origin sulphadoxine-resistant isolates in the parasite population. This could have caused the breakage in clonality of the single-origin sulphadoxine-resistant isolates and produced resistant isolates with multiple genetic backgrounds. Thereafter, the batches of resistant isolates possessing considerable genetic variations might have rapidly expanded in the parasite population for a short time. In other words, outcrossing that was sufficiently high to disrupt the clonality of the original sulphadoxine-resistant isolates might have continued since the emergence of resistant isolates. This is considered to be the main reason for the observed maintenance of within-population genetic variation. We observed that the number of loci with non-random associations in 2011 was smaller than that in 2010, which might support this scenario. In Papua New Guinea, outcrossing was estimated to be 52% in the early 1990s^26^ but, since then, no estimation has been reported. Instead, MOI has been widely used^5,6^ because of a strong association between MOI and the likelihood of outcrossing^27,28^. We obtained MOIs with 1.41 in 2010 and 1.35 in 2011, which are similar to previous estimations (1–2.12) in East Sepik Province in 2005 and in Madang Province in 2006^5^ and in a county-wide analysis (1–1.6) in 2008-2009^6^. These MOIs were much lower than those in African endemic regions such as Uganda where mean MOI was 4.46^29^. At this stage, there is no evidence that the observed degree of MOIs in Papua New Guinea is sufficient to quickly break the non-random association patterns. Even so, it is very likely that outcrossing played a considerable role in the persistence of high within-population genetic diversity observed in this study area.

Robust maintenance of genetic variation has been generally reported in malaria parasites. In western Kenya, significant decrease in transmission intensity after the implementation of insecticide-treated bed nets did not reduce the within-population genetic diversity in 10 years^30,31^. Similarly, genetic diversity at the border between Thailand and Myanmar remained stable throughout a 10-year study period, even though the incidence of *P*. *falciparum* infection decreased considerably from 0.24 per person per year in 2000 to 0.02 in 2010^8^. In Papua New Guinea, the first country-wide free distribution of LLIN was launched in 2004 with the financial support of round 3 and 8 grants from the Global Fund to Fight AIDS, Tuberculosis, and Malaria^19^. Through this distribution drive, significant increases in the ownership and use of LLINs were confirmed at sentinel surveillance sites^32^. A previous, well-designed cohort study in Papua New Guinea demonstrated a clear association between insecticide treated net usage and the reduction of prevalence, clinical episode, and MOIs^33^. Accordingly, the average population prevalence of falciparum malaria reduced from 10.1% pre-LLIN to 2.5% post-LLIN at the country-wide sentinel sites^21^. Despite these changes, however, the persistence of high-levels of within-population genetic diversity was confirmed by the analysis of neutral microsatellites^17^ and merozoite surface protein 2^5,6^. In our study area, LLINs were distributed in the early phases of the drive, just after our initial sampling in 2002 and 2003. Decreases in the prevalence of falciparum malaria and mean MOI were observed in 2010 and 2011, which indicates that malaria control measures performed between our sampling times had a considerable impact on the reduction of malaria. However, within-population genetic diversity continued to be high in our study site. Consistent with these observations, we have previously demonstrated that the physical distance of a population from a sub-Saharan African origin could be the primary reason for within-population diversity, rather than regional variations in transmission intensity and previous malaria-control interventions^34–36^.

In the present study, all parasites showed sulphadoxine-susceptible *dhps* alleles when sulphadoxine/pyrimethamine was introduced as the first-line treatment in the early 2000s. In contrast, approximately 70% of parasites already harboured the pyrimethamine-resistant *dhfr* allele (CNRNI). Similar results were previously shown in East Sepik^37,38^ and Madang province^37^. Historically, pyrimethamine alone was used as a prophylactic against malaria, as well as a drug to treat malarial infections in the 1950s^39^, this might have led to the selection of the pyrimethamine-resistant allele. The sulphadoxine-resistant SGEAA allele emerged between 2003 and 2010 in the present study area. This finding coincides with previous observations in Madang Province, where this allele appeared between 2006 and 2010^40–42^. However, frequencies of the SGEAA allele in our study (84% in 2011) were much higher than those in the previous studies (~25%)^40–42^. Importantly, despite the selection of the double mutant SGEAA allele, there is no evidence of the emergence of higher sulphadoxine-resistant alleles with three or more mutations in Papua New Guinea^40–42^, although these are highly prevalent in Southeast Asia^43^ and were recently observed in Africa^44^. Together with the observation that the highest pyrimethamine-resistance (I164L) is also absent in Papua New Guinea^45^, sulphadoxine/pyrimethamine efficacy is still retained against *P*. *falciparum*, as shown in a previous large-scale randomized trial on intermittent preventive treatment in infants^40,46^.

In conclusion, we report that a rapid expansion of sulphadoxine-resistant parasites did not result in the emergence of distinct parasite subpopulations, nor did it reduce the within-population genetic variation in the parasite population within our study area in Papua New Guinea; this is different from the patterns observed in the Greater Mekong Subregion where transmission intensity is much lower than that in Papua New Guinea^14,15^. Within-population genetic diversity reflects the potential of a population to survive in a constantly changing environment. In parasite populations with low levels of genetic diversity, short-term evolutionary changes to surmount various environmental challenges are not expected. Clonal expansions of drug-resistant parasites can potentially affect the genetic diversity of malarial parasite populations largely through their effects on local malaria ecology, such as levels of multiple-clonal infection and parasite migration. This emphasizes the importance of further studies in regions with variable local malaria ecology to investigate how rapid expansion of drug-resistant parasites can affect within-population genetic diversity.

## Materials and Methods

### Study site and parasite isolates

This study was conducted in a single catchment area that included 14 villages in the Boikin-Dagua Rural Local-Level Government area, East Sepik, Papua New Guinea (Fig. 1). All studied villages were located within 12 km west and 3 km east of a health centre in the catchment area. Most of the area included in this study was situated in a lowland swamp along the coast and experienced high transmission rates of malaria throughout the year despite seasonal fluctuations^47^. *P*. *falciparum* was the predominant malarial parasite species in the study area at the time of our surveys. The main vectors of malaria transmission were *Anopheles punctulatus*, *An*. *farauti*, and *An*. *koliensis*^20,47^. Entomological inoculation rates with *P*. *falciparum* calculated from a previous study in Dreikikir, a region ~50 km from our study area, were 159 infective bites/person/year in 2008 (before the long-lasting insecticidal net distribution) and 53 infective bites/person/year in 2011 (two years after distribution of long-lasting insecticidal nets)^20^. Sulphadoxine/pyrimethamine along with one of the 4-aminoquinolines (chloroquine or amodiaquine) was introduced as a first-line treatment for uncomplicated malaria in the early 2000s. Before its introduction as a first-line treatment, sulphadoxine/pyrimethamine was used as second-line treatment in combination with quinine.

We conducted two surveys in February 2002 and July 2003, immediately after sulphadoxine/pyrimethamine introduction, as well as two surveys conducted later in January–February of 2010 and 2011. Individuals living in the studied villages were asked to voluntarily participate in the surveys. Finger-prick blood samples were taken after obtaining informed consent for study participation from individuals or their guardians. Finger-prick blood samples (100 μL) from individuals were spotted onto chromatography filter papers (ET31CHR; Whatman Limited, Kent, UK). Parasite DNA was purified from a quarter of each blood spot using the QIAamp DNA Blood Mini Kit (QIAGEN, Hilden, Germany) according to a modified version of the manufacturer’s instructions^48^. We performed species-specific polymerase chain reactions (PCRs) to confirm *P*. *falciparum* infections as previously described^49^. This method is based on the amplification of 18S small subunit ribosomal RNA with a semi-nested, multiplex PCR. The first PCR amplification detects the *Plasmodium* genus in samples and the second amplification differentiates between the *Plasmodium* species that affect humans.

Ethical clearance for the study was obtained from the Medical Research Advisory Committee of the Papua New Guinea National Department of Health (No. 0.1.16 and 09.26), the Tokyo Women’s Medical University Ethical Committee (No. 1744), and the Juntendo University Ethical Committee (No. 2013009). All experiments were carried out in accordance with the approved guidelines.

### Haplotyping of ***pfcrt, dhfr***, and ***dhps*** and flanking microsatellites

The *P*. *falciparum* genes responsible for resistance to chloroquine (*pfcrt*), pyrimethamine (*dhfr*), and sulphadoxine (*dhps*) were sequenced as previously described^22,50,51^. Briefly, nested PCRs were performed to amplify sequences, including polymorphic sites known to confer resistance (i.e. amino acid positions 72–76 in *pfcrt*^52^, 50, 51, 59, 108, and 164 in *dhfr*^53^, and 436, 437, 540, 581, and 613 in *dhps*^54,55^), followed by direct sequencing using the BigDye Terminator v1.1 Cycle Sequencing Kit on an ABI PRISM 377 DNA Sequencer (both Applied Biosystems, Carlsbad, CA, USA). Allele frequencies of drug-resistance genes were estimated using MalHaploFreq^23^; the program utilizes allele prevalence and MOI data to estimate allele frequencies with a maximum likelihood algorithm using maximum likelihood methodology. *P*-value < 0.05 was considered significant unless otherwise specified.

Alleles of the six microsatellite markers located near the *dhps* gene (−2.9 kb, −1.5 kb, −0.13 kb, +0.8 kb, +4.3 kb, and +7.7 kb) were determined to investigate if mutant (sulphadoxine-resistant) alleles in the studied population had a single origin or multiple origins as previously reported^56,57^. We have previously demonstrated that very limited origins of resistance for chloroquine and pyrimethamine resistance (one in a resistant *pfcrt* haplotype and two in resistant *dhfr* haplotypes) had spread in the studied area^51,58^. Semi-nested PCR products with fluorescent end-labelled primers were separated on an GenomeLab GeXP (Beckman Coulter, Brea, CA, USA) and size variations were determined with the GenomeLab Genetic Analysis System (Beckman Coulter). If minor peak heights were greater than one third of the major peak height, these peaks were regarded as peaks from minor clones. Although this one-third criterion potentially underestimates minor alleles with peak heights lower than 33% of the dominant clone, mis-scoring of artefacts caused by stutter peaks and other technical errors can be largely avoided, because of which, this method is widely used^59,60^.

### Neutral microsatellite genotyping and MOI

Nucleotide length variations were measured in 10 putatively neutral microsatellite markers: TA42 (chromosome 5), TA81 (chromosome 5), TA1 (chromosome 6), TA109 (chromosome 6), TA87 (chromosome 6), 2490 (chromosome 10), ARA2 (chromosome 11), Pfg377 (chromosome 12), PfPK2 (chromosome 12), and TA60 (chromosome 13)^3,25,61^. These markers are located on different chromosomes from *pfcrt* (chromosome 7), *dhfr* (chromosome 4), and *dhps* (chromosome 8), and are commonly used for population genetic analyses of malarial parasites. Microsatellite alleles for the 10 putatively neutral microsatellites were detected and scored with the same methodology described in the previous section.

Samples harbouring one or more loci with multiple alleles were interpreted as multiple-clonal infections. The greatest number of alleles at the most polymorphic locus was used to determine the multiplicity of infection (MOI) or the number of clones per sample^59,62^. Since this method cannot reconstruct the multilocus genotype of each clone, MOI measures only provide the minimum number of clones per isolate.

### Population structure

Standardized data on 10 putatively neutral microsatellite markers were subjected to principal component analysis (PCA). Genotype data were represented by a matrix *G* with elements *g*_*ij*_ corresponding to isolate *i* (*i* = 1, …, *n*) and microsatellite locus *j* (*j* = 1, …, *m*). The genotype value at a microsatellite locus was initially specified as the nucleotide length of the predominant allele. Next, the genotype entries were standardized for each microsatellite locus by replacing *g*_*ij*_ with

1 $${x}_{ij}\,=\,({g}_{ij}\,-\,\overline{{g}_{j}})/{\sigma }_{j}$$

where,

2 $$\overline{{g}_{j}}\,=\,\sum _{i\,=\,1}^{n}{g}_{ij}/n$$

and,

3 $${\sigma }_{j}\,=\,\sqrt{\sum _{i\,=\,1}^{n}{({g}_{ij}-\overline{{g}_{j}})}^{2}/n}$$

denote the mean and standard deviation of the genotype value at microsatellite locus *j* respectively. Since the PCA does not allow missing data, only isolates without missing genotypes at 10 putatively neutral microsatellite loci were used in the calculation of *D*_*ij*_. In multi-clonal infections, major alleles were used for the analysis. We also used STRUCTURE 2.3.4, a Bayesian model-based clustering program, to calculate the most probable number of clusters (K) in the sample set and then assign probabilities of membership to each cluster for each individual^63^. For each run, a burn-in period of 50,000 steps was followed by 1 × 10^5^ iterations under the no admixture model and the assumption of correlated allele frequencies among populations. For each K of 1–10 clusters, 10 runs were performed. ΔK was calculated as previously described^64^. A visual output for the run of highest Ln P(D) was generated using the program Distruct^65^.

### Genetic diversity

Several population genetic measurements were used to estimate the extent of genetic diversity in the parasite population according to the nucleotide-length variations in 10 putatively neutral microsatellite markers. In case of multiple-clonal infections, major alleles were used. These measurements were: number of alleles, allelic richness, and expected heterozygosity; expected heterozygosity is defined as the probability that two randomly selected isolates have different microsatellite alleles in the population and is calculated for each microsatellite locus using the following formula:

4 $$h=1-\sum _{i=1}^{n}{p}_{i}^{2}$$

where *n* represents the total number of malaria isolates, and *p*_*i*_ is the frequency of the *i*th microsatellite allele. The allele frequencies were obtained by counting only predominant alleles (i.e. one allele from each isolate). To assess the differences in expected heterozygosity between groups, a permutation test with 1,000 random shuffles was conducted for each microsatellite locus. In this permutation test, the collection-year label was randomly shuffled to obtain a distribution of differences in expected heterozygosity between two groups under the null hypothesis. Other measurements (proportion of polymorphic loci and number of alleles) were determined using FSTAT version 2.9.3^66^.

### Non-random association

Estimations of pairwise non-random associations between 10 putatively neutral microsatellite loci, *pfcrt*, *dhfr*, and *dhps* were carried out using Genepop version 4.1 under the following Markov chain parameters: dememorization number = 1000, number of batches = 100, and number of iterations per batch = 1000^67^. A predominant allele was considered for each microsatellite locus. Bonferroni-corrected *P* value threshold was used to adjust for multiple comparisons.

The variance of the pairwise differences for the observed data (*V*_D_) and the variance expected for linkage equilibrium (*V*_e_) were computed using LIAN 3.7 programme^68^. A standardized measure of linkage, *I*^*S*^_*A*_ was calculated as

5 $${I}_{A}^{S}=\frac{1}{l-1}(\frac{{V}_{D}}{{V}_{E}}-1)$$

where *l* is the number of loci tested. In the calculation, only isolates without missing genotypes at the 10 putatively neutral microsatellite loci were used. To further evaluate the null hypothesis that all these loci are in linkage equilibrium (i.e., complete interlocus independence), Monte-Carlo method was implemented in LIAN 3.7 programme^68^. In this test, the data set was shuffled 10,000 times by resampling the loci without replacement, and the variance for each resampled data set was compared with *V*_D_. The *P*-value was defined as the frequency with which the variance for the resampled data set was greater or equal to *V*_D_ (i.e., one-sided test).

## Electronic supplementary material

Supplementary Information
