# Supplementary material for: Rapid selection of sulphadoxine-resistant Plasmodium falciparum and its effect on within-population genetic diversity in Papua New Guinea
Source: Sci Rep. 2018 Apr 3;8:5565. doi: 10.1038/s41598-018-23811-7 (PMC5882878; doi:10.1038/s41598-018-23811-7)

Supplementary Information

Title:

Rapid selection of sulphadoxine-resistant Plasmodium falciparum and its effect on within-population genetic diversity in Papua New Guinea

Full author list:

Toshihiro Mita^1,*^, Francis Hombhanje^2^, Nobuyuki Takahashi^3^, Makoto Sekihara^1^, Masato Yamauchi^1^, Takahiro Tsukahara^3^, Akira Kaneko^4,5^, Hiroyoshi Endo^3^, and Jun Ohashi^6^

^1^ Department of Tropical Medicine and Parasitology, Juntendo University School of Medicine, 2-1-1 Hongo, Bunkyo, Tokyo, 113-8421, Japan

^2^ Centre for Health Research & Diagnostics, Divine Word University, P.O. Box 483, Madang, Papua New Guinea

^3^ Department of International Affairs and Tropical Medicine, Tokyo Women’s Medical University, 8-1 Kawada-cho, Shinjuku-ku, Tokyo 162-8666, Japan.

^4^ Department of Microbiology, Tumor and Cell Biology, Karolinska Institutet, SE-171 77 Stockholm, Sweden

^5^ Department of Parasitology, Osaka City University Graduate School of Medicine, Asahi-cho 1-4-3, Abeno-ku, Osaka, 545-8585, Japan

^6^ Department of Biological Sciences, Graduate School of Science, The University of Tokyo, Tokyo, Japan, 7-3-1 Hongo, Bunkyo-ku, Tokyo 113-0033, Japan

* Corresponding author; [tmita@juntendo.ac.jp](mailto:tmita@juntendo.ac.jp)

**Supplementary Fig. S1.** **Haplotyping of *pfcrt, dhfr, dhps,* and 10 microsatellite loci in 302 *Plasmodium falciparum* isolates obtained in 2002, 2003, 2010, and 2011.** Capital letters denote amino acid residues at positions 72–76 in *pfcrt*; 50, 51, 59, 108, and 164 in *dhfr*; and 436, 437, 540, 581, and 613 in *dhps*; mutations are underlined. Undetermined haplotypes or microsatellites are shown as ND.

| Year | *pfcrt* | *dhfr* | *dhps* | ARA2 | Pfg377 | TA81 | TA60 | TA1 | TA42 | TA109 | TA87 | 2490 | PfPK2 |
| --- | --- | --- | --- | --- | --- | --- | --- | --- | --- | --- | --- | --- | --- |
| 2002 | SVMNT | CNRNI | SAKAA | 72 | 95 | 132 | 87 | 168 | 188 | 163 | 101 | 85 | 170 |
|  | CVMNK | CNCSI | SAKAA | 75 | 98 | 132 | 87 | 183 | 188 | 163 | 101 | 82 | 170 |
|  | SVMNT | CNCSI | SAKAA | 72 | 95 | 132 | 87 | 171 | 191 | 163 | 107 | 85 | 185 |
|  | SVMNT | CNRNI | SAKAA | 78 | 104 | 129 | 84 | 177 | 188 | 163 | 104 | 82 | 170 |
|  | SVMNT | CNRNI | SAKAA | 75/72 | 98 | 132/123 | 84 | 171 | 188 | 163 | 107 | 85/82 | 167 |
|  | SVMNT | CNCSI | SAKAA | 78 | 95 | 132 | 93 | 180 | 188 | 163 | 107/104 | 82 | 176/173 |
|  | SVMNT | CNRNI | SAKAA | 69 | 95 | 132/123 | 90 | 186 | 188 | 163 | 101 | 82 | 182/170 |
|  | SVMNT | CNRNI | SAKAA | 81 | 95 | 129/132 | 87 | 177 | 188 | 163 | 107/95 | 82 | 170 |
|  | SVMNT | CNRNI | SAKAA | 69 | 98 | 123/132 | 90 | 180 | 188 | 163 | 107/104 | 82 | 167 |
|  | SVMNT | CNRNI | SAKAA | 72 | 95/98 | 132/117 | 87 | 177 | 185 | 163 | 104 | 82 | 173 |
|  | SVMNT | CNRNI | SAKAA | 72 | 101/95 | 132/120 | 87/81 | 168/177 | 188 | 163/169 | 101/107 | 85/82 | 170/179 |
|  | CVMNK | CNRNI | SAKAA | 72 | 95 | 132/117 | 84 | 174 | ND | 163 | 104/101 | 82 | 197 |
|  | SVMNT | CNRNI | SAKAA | 75 | 95 | 120 | 87 | 168 | 188 | 163 | 107 | 85 | 173 |
|  | SVMNT | CNCSI | SAKAA | 75 | 98 | 123/132 | 78 | 171 | 188 | 163 | 104 | 85 | 185 |
|  | SVMNT | CNRNI | SAKAA | 69 | 95/104 | 132 | 87 | 162 | ND | 163 | 113 | 82/70 | 176/173/185 |
|  | SVMNT | CNRNI | SAKAA | 72 | 98 | 126 | 84 | 168 | 188 | 187 | 104 | 88 | 170 |
|  | SVMNT | CNRNI | SAKAA | 81 | 95 | 129 | 87 | 168 | 188 | 163 | 104 | 82 | 182 |
|  | SVMNT | CNRNI | SAKAA | 72 | 98 | 123 | 87 | 174 | 188 | 163 | 104 | 82 | 173 |
|  | SVMNT | CNRNI | SAKAA | 72 | 95 | 129 | 84 | 177 | 188 | 163 | 104 | 82 | 173 |
|  | SVMNT | CNCSI | SAKAA | 69 | 104 | 117 | 90 | 174 | 191 | 163 | 101 | 82 | 170 |
|  | SVMNT | CNRNI | SAKAA | 69 | 95 | 117 | 87 | 171 | 188 | 163 | 101 | 82 | 170 |
|  | SVMNT | CNRNI | SAKAA | 84 | 101 | 132/129 | 87 | 171 | 188 | 163 | 101 | 82 | 173 |
|  | SVMNT | CNCSI | SAKAA | 69 | 98 | 120 | 81 | 168 | 188 | 163 | 110 | 82 | 170 |
|  | SVMNT | CNCSI | SAKAA | 72 | 95 | 132/120 | 87 | 171 | 206 | 163 | 101 | 82 | 173 |
|  | SVMNT | CNCSI | SAKAA | 72 | 98 | 120 | 87 | 174 | 188 | 163 | 104 | 82 | 194 |
|  | SVMNT | CNRNI | SAKAA | 72 | 98 | 132/129 | 87 | 168/171 | 188 | 163 | 104/107/101 | 82 | 179/173/185 |
|  | SVMNT | CNCSI | SAKAA | 75 | 98 | 129 | 84 | 165 | 188 | 163 | 101 | 82 | 176 |
|  | SVMNT | CNCSI | SAKAA | 78 | 98 | 129 | 99 | 177 | 188 | 178 | 107 | 82 | 170 |
|  | SVMNT | CNRNI | SAKAA | 72/69 | 98/104 | 129 | 84 | 174 | 188 | 163 | 101 | 85/82 | 170 |
|  | CVMNK | CNCSI | SAKAA | 69 | 95 | 132 | 87 | 168 | 188 | 163 | 110 | 82 | 173 |
|  | SVMNT | CNRNI | SAKAA | 69 | 98 | 135 | 87 | 168 | 191 | 163 | 104 | 91 | 182 |
| 2003 | SVMNT | CNCSI | SAKAA | 72/84/69 | 98/95/101 | 120 | 87 | 168 | 188 | 163 | 101/104 | 82/85 | 170/173 |
|  | SVMNT | CNRNI | SAKAA | 81 | 95 | 120 | 84 | 171 | 188 | 163 | 104 | 82 | 167 |
|  | SVMNT | CNRNI | SAKAA | 72/69 | 98/95/101 | 132 | 84 | 174/171 | 188 | 163/172 | 104/107 | 73 | 170/173/176 |
|  | SVMNT | CNRNI | SAKAA | 69 | 95 | 126 | 90 | 168 | 188 | 163 | 107 | 85 | 164 |
|  | SVMNT | CNRNI | SAKAA | 84 | 98 | 117 | 84 | 168 | 188 | 163 | 101 | 82 | 170 |
|  | SVMNT | CNRNI | SAKAA | 75 | 98 | 132 | 87 | 183 | 188 | 163 | 107 | 82 | 170 |
|  | SVMNT | CNRNI | SAKAA | 69 | 98 | 123 | 84 | 171 | 188 | 169 | 104 | 82 | 173 |
|  | SVMNT | CNCSI | SAKAA | 81 | 95 | 132 | 87 | 171 | 188 | 163 | 101 | 82 | 176 |
|  | SVMNT | CNRNI | SAKAA | 72 | 95 | 126/132 | 84 | 186 | 188 | 163 | 104 | 82 | 170 |
|  | SVMNT | CNRNI | SAKAA | 69 | 98 | 117/132 | 93/84 | 171/168 | 188/185 | 163 | 107 | 82 | 167 |
|  | SVMNT | CNRNI | SAKAA | 69 | 95 | 132 | 84/93 | ND | 188 | 163 | 107 | 82 | 167 |
|  | SVMNT | CNRNI | SAKAA | 75 | 98 | 132/126 | 87 | 183 | 188 | 163 | 107 | 73 | 170 |
|  | SVMNT | CNRNI | SAKAA | 72 | 95 | 117 | 90 | 171 | 188 | 163 | 101 | 82 | 179 |
|  | SVMNT | CNRNI | SAKAA | 81 | 98 | 129 | 84 | 171 | 191 | 166 | 107 | 82 | 173 |
|  | SVMNT | CNRNI | SAKAA | 60 | 98 | 132 | 87 | 174 | 188 | 163 | 98 | 82 | 170 |
|  | SVMNT | CNRNI | SAKAA | 69 | 101 | 132 | 87 | 168 | 188 | 163 | 110 | 82 | 179 |
|  | SVMNT | CNRNI | SAKAA | 72 | 95 | 123/132 | 75 | 165 | 188 | 163 | 101 | 82/79 | 182 |
|  | SVMNT | CNRNI | SAKAA | 78 | 98 | 120 | 84 | 174 | 188 | 163 | 107 | 82 | 170 |
|  | SVMNT | CNRNI | SAKAA | 72/78 | 101/98/110 | 132 | 93 | 171 | 188 | 163 | 101 | 82 | 185/176/173 |
|  | SVMNT | CNRNI | SAKAA | 78 | 101 | 132 | 87 | 183 | 188 | 163 | 107 | 82 | 164 |
|  | SVMNT | CNCSI + CNCNI | SAKAA | 72/78 | 95/98 | 132/119 | 87 | 171 | 191/188 | 166/163 | 104/107 | 82 | 179/176/173 |
|  | SVMNT | CNRNI | SAKAA | 69 | 101 | 132 | 87 | 168 | 188 | 163 | 110 | 82 | 179 |
|  | SVMNT | CNRNI | SAKAA | 69 | 95 | 132/120 | 87 | 165 | 188 | 163 | 107 | 85 | 173 |
|  | SVMNT | CNCSI | SAKAA | 75 | 98 | 126/132 | 90 | 171 | 191 | 163 | 113 | 91 | 170 |
|  | SVMNT | CNRNI | SAKAA | 72 | 98 | 138 | 84 | 171 | 188 | 163 | 104 | 82 | 170 |
|  | SVMNT | CNRNI | SAKAA | 69 | 104 | 123/132 | 84 | 174 | 188 | 163 | 101 | 82 | 170 |
|  | SVMNT | CNRNI | SAKAA | 75 | 98 | 132 | 87 | 183 | 188 | 163 | 107 | 82 | 170 |
|  | SVMNT | CNRNI | SAKAA | 69 | 98 | 132 | 90 | 174 | 191 | 163 | 98 | 82 | 167 |
|  | SVMNT | CNRNI | SAKAA | 78 | 98 | 123/132 | 87 | 171 | 188 | 163 | 107 | 85 | 170 |
|  | SVMNT | CNRNI | SAKAA | 72/81 | 98 | 116 | 84/87 | 171 | 188 | 163 | 104 | 82/91 | 173/176 |
|  | SVMNT | CNRNI | SAKAA | 78/72 | 98/101 | 132/129 | 87 | 171/168 | 188 | 163 | 104/101 | 82/79 | 164 |
|  | SVMNT | CNRNI | SAKAA | 81/78 | 98/110 | 132 | 87 | 171 | 188 | 163 | 107 | 85 | 185 |
|  | SVMNT | CNRNI | SAKAA | 60 | 98 | 132 | 93 | 177 | 188 | 163 | 101 | 82 | 179 |
|  | SVMNT | CNCSI | SAKAA | 69 | 98 | 138 | 87 | 171 | 188 | 163 | 101 | 82 | 173 |
|  | SVMNT | CNCSI + CNCNI | SAKAA | 69/72 | 95/101 | 132/129 | 87/90/84 | 176 | 188/185 | 163 | 101 | 85/82 | 176/170/167 |
|  | SVMNT | CNCSI | SAKAA | 69 | 98 | 132 | 87 | 168 | 188 | 163 | 101 | 82 | 176 |
|  | SVMNT | CNCSI | SAKAA | 66 | 98 | 126 | 87 | 189 | 188 | 163 | 104 | 82 | 170 |
|  | SVMNT | CNRNI | SAKAA | 75/69 | 98 | 120/132 | 84 | 168 | 188 | 151/163 | 107 | 82 | 173/170 |
|  | SVMNT | CNRNI | SAKAA | 72 | 95 | 132/129 | 87/84 | 177 | 188 | 163 | 98/104/101 | 85 | 173 |
|  | SVMNT | CNRNI | SAKAA | 72 | 98 | 117 | 84 | 177 | 188 | 163 | 104 | 82 | 173 |
|  | SVMNT | CNRNI | SAKAA | 69 | 95 | 126 | 90 | 168 | 188 | 163 | 107 | 85 | 164 |
|  | SVMNT | CNCNI | SAKAA | 69 | 98 | 132 | 87 | 168 | 188 | 163 | 101 | 82 | 176 |
|  | SVMNT | CNRNI | SAKAA | 75/69/72 | 98/110 | 132 | 84/90/87 | ND | 188/185 | 163 | 107/98 | 82 | 170/185/167 |
|  | SVMNT | CNCSI | SAKAA | 69/72 | 98 | 132/120 | 93/84 | 171 | 188 | 163 | 107/104 | 82 | 167 |
|  | SVMNT | CNRNI | SAKAA | 69 | 104/101 | 123/132 | 84 | 168 | 188/185 | 163 | 110 | 82 | 170 |
|  | SVMNT | CNRNI | SAKAA | 84 | 95 | 132 | 87 | 180 | 188 | 163 | 107 | 82 | 170 |
|  | SVMNT | CNRNI | SAKAA | 69/78 | 95 | 123/129 | 87 | 168 | 188 | 163 | 101/107 | 85/82 | 170 |
|  | SVMNT | CNRNI | SAKAA | 69 | 95 | 132 | 87 | 171 | 203 | 163 | 104 | 82 | 170 |
|  | SVMNT | CNRNI | SAKAA | 72/69/78 | 95/98 | 132/129/117 | 87/84 | 177 | 188 | 163 | 98/95/104 | 85 | 182 |
|  | SVMNT | CNRNI | SAKAA | 72 | 98 | 117 | 84 | 177 | 188 | 163 | 104 | 82 | 173 |
|  | SVMNT | CNRNI | SAKAA | 69 | 95/98 | 129/132/117 | 87/90 | 171/168 | 188/185 | 163 | 113/107/110 | 82 | 173 |
|  | SVMNT | CNRNI | SAKAA | 69 | 98 | 120 | 87 | 177 | 188 | 163 | 110 | 82 | 170 |
|  | SVMNT | CNRNI | SAKAA | 78 | 95 | 123 | 84 | 168 | 188 | 163 | 107 | 82 | 173 |
|  | SVMNT | CNRNI | SAKAA | 69 | 104 | 123 | 84 | 174 | 188 | 163 | 101 | 82 | 170 |
|  | SVMNT | CNRNI | SAKAA | 75/72 | 98 | 132 | 87 | 171/176 | 191/188 | 163 | 104/107 | 82/85 | 185/164 |
| 2010 | SVMNT | CNRNI | SAKAA | 75 | 98 | 123 | 87/75 | 174 | 191 | 163 | 101 | 82 | 185/206 |
|  | SVMNT | CNRNI | SGEAA | 81 | 98 | 120 | 87 | 165 | 188 | 163 | 107 | 79 | 173 |
|  | SVMNT | CNRNI | SAKAA | 75 | 98 | 123 | ND | 168 | 188 | 163 | 101 | 82 | 185 |
|  | SVMNT | CNRNI | SAKAA | 69 | 98 | 132 | 84 | 171 | 188 | 163 | 107 | 82 | 170 |
|  | SVMNT | CNRNI | SGEAA | 69 | 98 | 120 | 87 | ND | 188 | 163 | 107 | 82 | 179 |
|  | SVMNT | CNRNI | SAKAA | 69 | 98 | 132 | ND | 171 | 188 | 163 | 77 | 82 | ND |
|  | SVMNT | CNRNI | SGEAA | 69 | 98 | 120/132 | 99/96 | 186 | 191 | 163 | 98 | 82 | 170 |
|  | SVMNT | CNRNI | SAKAA | 75 | 95 | 123/132 | 84 | 174 | 188 | 163 | 107/104 | 85 | 170 |
|  | SVMNT | CNRNI | SGKAA | 72 | 95 | 123 | 87 | 174 | 188 | 163 | 98 | 82 | 176 |
|  | SVMNT | CNRNI | SAKAA | 69 | 98 | 132 | 84 | 171 | 188 | 163 | 107 | 82 | 170 |
|  | SVMNT | CNRNI | SAKAA | 69 | 98 | 120 | 84 | 177 | 188 | 163 | 107 | 82 | 170 |
|  | SVMNT | CNRNI | SGEAA | 81/78 | 98 | 123 | 87 | 165 | 188 | 163 | 113 | 82 | 176 |
|  | SVMNT | CNRNI | SGEAA | 81/78 | 98 | 123 | 87 | 171 | 188 | 163 | 113 | 85 | 176 |
|  | SVMNT | CNRNI | SGEAA | 81 | 101 | 123 | 87 | 165 | 188 | 163 | 113 | 82 | 176 |
|  | SVMNT | CNRNI | SGEAA | 72 | 98 | 120 | 81 | 168 | 188 | 163 | 113 | 82 | 170 |
|  | SVMNT | CNRNI | SAKAA | ND | ND | 132 | 84 | ND | 185/188 | 163 | 107 | 82 | 191 |
|  | SVMNT | CNRNI | SGEAA | 81/78 | 98 | 120 | 84 | 168 | 188 | 163 | 101 | 82 | 173 |
|  | SVMNT | CNRNI | SAKAA | 69 | 95 | 120/132 | 87 | 171 | 188 | 160 | 107 | 82 | 173 |
|  | SVMNT | CNRNI | SGEAA | 93/90 | 98 | 129 | 87 | 177 | 188 | 160 | 107 | 82 | 170 |
|  | SVMNT | CNRNI | SGEAA | 75 | 98 | 120 | 84 | 174 | 188 | 163 | 107 | 82 | 173 |
|  | SVMNT | CNRNI | SAEAA | 81/78 | 98 | 132 | 87 | 168 | 188 | 163 | 113 | 82 | 176 |
|  | SVMNT | CNRNI | SGEAA | 69 | 98 | 132 | 84 | 168 | 188 | 163 | 101 | 82 | 173 |
|  | SVMNT | CNRNI | SGEAA | 72/69 | 95 | 120 | 87 | 174 | 188 | 163 | 95 | 82 | 176 |
|  | SVMNT | CNRNI | SGEAA | 75 | 98 | 132 | 84 | 174 | 188 | 163 | 107 | 82 | 173 |
|  | SVMNT | CNRNI | SGEAA | 81 | 101 | 126 | 81 | 171 | 188 | 163 | 104 | 82 | 206 |
|  | SVMNT | CNRNI | SAKAA | 72 | 98 | 123 | 84 | 168 | 188 | 163 | 95 | 82 | 170 |
|  | SVMNT | CNRNI | SAKAA | 75 | 98 | 123 | 87 | 168 | 188 | 163 | 98 | 82 | 206 |
|  | SVMNT | CNRNI | SGEAA | 75 | 98 | 120 | 84 | 168 | 185 | 160 | 107 | 82 | 173 |
|  | SVMNT | CNRNI | SAKAA | ND | ND | 132 | 84/96 | 177 | 188 | 163 | 104 | 82 | 170/188 |
|  | SVMNT | CNRNI | SAKAA | 66 | 98 | 120 | 84 | 171 | 188 | 160 | 107 | 82 | 170 |
|  | SVMNT | CNRNI | SAKAA | 75/69 | 95 | 123 | 90 | 174 | 188 | 163 | 104 | 82 | 170 |
|  | SVMNT | CNRNI | SGKAA | ND | 95 | 132 | 84 | 168 | 191 | 163 | 98 | 91 | 176 |
|  | SVMNT | CNRNI | SGEAA | 69 | 98 | 132 | ND | ND | 185 | 163 | 98 | 82 | 185 |
|  | SVMNT | CNRNI | SGEAA | 69 | 98 | 120 | 99 | 168 | 188 | 163 | 98 | 82 | 179 |
|  | SVMNT | CNRNI | ND | 69 | 98 | 120 | 84 | 171 | 188 | 160 | 107 | 82 | 170 |
|  | SVMNT | CNRNI | SAKAA | 69 | 98 | 120 | 84 | 171 | 188 | 163 | 107 | 82 | 170 |
|  | SVMNT | CNRNI | SGEAA | 69/75 | 98 | 132/119 | 84 | 168 | 188 | 163 | 107 | 82 | 173/179 |
|  | SVMNT | CNRNI | SGEAA | 69 | 98 | 120 | 99 | 168 | 188 | 163 | 98 | 82 | 179 |
|  | SVMNT | CNRNI | SGEAA | 69 | 95 | 132 | ND | 168 | 185 | 163 | 104 | 85 | 188 |
|  | SVMNT | CNRNI | SGEAA | 69 | 98 | 132 | 84 | 171 | 188 | ND | 107 | 82 | 188/170 |
|  | SVMNT | CNRNI | ND | 66 | 95 | 132 | 84 | 171 | 188 | 169 | 104 | 82 | 173 |
|  | SVMNT | CNRNI | SGEAA | 72/81 | 95 | 132 | 87 | ND | 179 | 163 | 107 | 82 | 173 |
|  | SVMNT | CNRNI | SAKAA | 72 | 95 | 132/120 | 78 | ND | 188 | 163 | 98 | 82 | 173/167/185 |
|  | SVMNT | CNRNI | SGEAA | ND | 98 | 132 | 84 | ND | 188 | 163 | 101 | 82 | 173 |
|  | SVMNT | CNRNI | SAKAA | 69 | 98 | 120 | 84 | 171 | 188 | 163 | 107 | 82 | 170 |
|  | SVMNT | CNRNI | SGEAA | 69 | 98 | 120 | 99 | 168 | 188 | 163 | 98 | 82 | 179 |
|  | SVMNT | CNRNI | SGEAA | 93 | 98 | 129 | 87 | 171 | 188 | 163 | 107 | 85 | 170 |
|  | SVMNT | CNRNI | SAKAA | 84 | 98 | 132 | ND | ND | 188 | ND | 101 | 82/96 | ND |
|  | SVMNT | CNRNI | SAKAA | 69 | 98 | 123 | 72 | 174 | 188 | 163 | 104 | 82 | 170 |
|  | SVMNT | CNRNI | SGEAA | 75 | 98 | 120 | 78 | 165 | 188 | 163 | 107 | 82 | 173 |
|  | SVMNT | CNRNI | SAKAA | 69 | 98 | 132 | 84 | 171 | 188 | 163 | 107 | 85 | 170 |
|  | SVMNT | CNRNI | SGEAA | 81 | 98 | 123 | 87 | 165 | 188 | 163 | 113 | 82 | 176 |
|  | SVMNT | CNRNI | SAKAA | 75 | 98 | 132 | 87 | 168 | 188 | 163 | 104 | 82 | 173 |
|  | SVMNT | CNRNI | SGEAA | 81 | 95 | 132/126 | 96 | 177 | 188 | 163 | 104 | 82 | 185 |
|  | SVMNT | CNRNI | SGEAA | 81 | 95 | 123 | 87 | 165 | 188 | 163 | 107 | 85 | 173 |
|  | SVMNT | CNRNI | SAKAA | 69 | 98 | 120 | 84 | 171 | 188 | 163 | 107 | 82 | 170 |
|  | SVMNT | CNRNI | SAKAA | 69 | 98 | 120/132 | 84 | 171 | 188 | 163 | 107 | 82 | 170 |
|  | SVMNT | CNRNI | SAKAA | 69 | 98 | 132 | 84 | ND | 188 | 163 | 107 | 85 | 170/185 |
|  | SVMNT | CNRNI | SAKAA | 69 | 98/95 | 120 | 84 | 171 | 188 | 163 | 107 | 82 | 170 |
|  | SVMNT | CNRNI | SAKAA | 69 | 98 | 132 | 84 | 171 | 188 | 163 | 107 | 85 | 170 |
|  | SVMNT | CNRNI | SGEAA | 69/81 | 98 | 132/120 | 99 | 168 | 188 | 163 | 98/101 | 85 | 170/185 |
|  | SVMNT | CNRNI | SGEAA | 78 | 101 | 126 | 75 | 168 | 188 | 163 | 104 | 82 | 206 |
|  | SVMNT | CNRNI | SGEAA | 81 | 98 | 123 | 87 | 165 | 188 | 163 | 113 | 85 | 176 |
|  | SVMNT | CNRNI | SGEAA | 69/81 | 95 | 120 | 84 | 165/171 | 188 | 163 | 113 | 82 | 176 |
|  | SVMNT | CNRNI | SAKAA | 69 | 98 | 120 | 84 | 171 | 188 | 163 | 107 | 82 | 170 |
|  | SVMNT | CNRNI | SGEAA | 81 | 101 | 132 | 75 | 168 | 188 | 163 | 101 | 82 | 194 |
|  | SVMNT | CNRNI | SGEAA | 72 | 101 | 120 | 81/87 | 171/168 | 188/206 | 163 | 110 | 82 | 170 |
|  | SVMNT | CNRNI | SGEAA | 93 | 98 | 132 | 87 | 171 | 188 | 163 | 107 | 82 | 170 |
|  | SVMNT | CNRNI | SGEAA | 81 | 95 | 123 | 87 | 165 | 188 | 163 | 107 | 85 | 173 |
|  | SVMNT | CNRNI | SGEAA | 81 | 98 | 120 | 84 | 168 | 188 | 163 | 101 | 82 | 173 |
|  | SVMNT | CNRNI | SAKAA | 69 | 98 | 120 | 81 | 168 | 188 | 163 | 107 | 82 | 170 |
|  | SVMNT | CNRNI | SGEAA | 81 | 101 | 129 | 78 | 171 | 188 | 163 | 101 | 82 | 194 |
|  | SVMNT | CNRNI | SGEAA | 81 | 95 | 123 | 96/78 | 177 | 188 | 163 | 104 | 82 | ND |
|  | SVMNT | CNRNI | SGEAA | ND | 98 | 132 | 99 | ND | 188 | 163 | 104 | 82 | 185/179 |
|  | SVMNT | CNRNI | ND | 72 | 95 | 132 | 87 | 168 | 188 | 163 | 95 | 82 | 185 |
|  | SVMNT | CNRNI | SGEAA | 84 | 95 | 129 | 84 | 165 | 188 | 163 | 95 | 82 | 170 |
|  | SVMNT | CNRNI | SGEAA | 78 | 98 | 138 | 90 | 171 | 188 | 160 | 110 | 82 | 176 |
|  | SVMNT | CNRNI | SAKAA | 72 | 95 | 132 | 93 | 168 | 188 | 160 | 107 | 82 | 173 |
|  | SVMNT | CNRNI | SGEAA | 84/81 | 95 | 129 | 78 | 168 | 188 | 163 | 98/104/95 | 82 | 170 |
|  | SVMNT | CNRNI | SGEAA | 75 | 98 | 120 | 84 | 168 | 188 | 163 | 107 | 82 | 173 |
|  | SVMNT | CNRNI | SAKAA | 69 | 98 | 120/132 | 84 | 171 | 188 | 163 | 107 | 82 | 170 |
|  | SVMNT | CNRNI | SGEAA | 78 | 98 | 132 | 99 | 165 | 188 | 163 | 98 | 82 | 188 |
|  | SVMNT | CNRNI | SGEAA | 81 | 101 | 126 | 72 | 165 | 188 | 163 | 101 | 82 | 206 |
|  | SVMNT | CNRNI | SGEAA | 81 | 95 | 129 | 84 | 171 | 188 | 163 | 98 | 82 | 170 |
|  | SVMNT | CNRNI | SGKAA | 69 | 95 | 123 | 87/99 | ND | 188 | 163 | 95 | 82 | 176 |
|  | SVMNT | CNRNI | SAKAA | 72 | 98 | 123 | 84 | 174 | 188 | 163 | 95 | 82 | 170 |
|  | SVMNT | CNRNI | SGKAA | 72 | 95 | 123 | 87 | 174 | 188 | 163 | 98 | 82 | 176 |
|  | SVMNT | CNRNI | SGEAA | 75 | 95 | 132/129 | 78 | ND | 185/188 | 163 | 110 | 82 | 170/185 |
|  | SVMNT | CNRNI | SAKAA | 78 | 98 | 132 | 99 | 165 | 188 | 163 | 98 | 82 | 185 |
|  | SVMNT | CNRNI | SGEAA | 78 | 98 | 120 | 99 | 168 | 188 | 163 | 98 | 82 | 206 |
|  | SVMNT | CNRNI | SGEAA | 75 | 98 | 120 | 84 | 171 | 188 | 160 | 104 | 82 | 173 |
|  | SVMNT | CNRNI | SGEAA | 72 | 95 | 120 | 90 | 171 | 188 | 163 | 101 | 82 | 206 |
|  | SVMNT | CNRNI | SGEAA | 75 | 98 | 120 | 84 | 168 | 188 | 163 | 104 | 82 | 173 |
|  | SVMNT | CNRNI | SAKAA | 75 | 98 | 132 | 84 | 168 | 185 | 163 | 107 | 85 | 185 |
|  | SVMNT | CNRNI | SAKAA | 75 | 95 | 138 | 87 | 168 | 188 | 151 | 95 | 82 | 173 |
|  | SVMNT | CNRNI | SAKAA | 69 | 98 | 120/134 | 84 | 171 | 188 | 163 | 107 | 82 | 170/185 |
|  | SVMNT | CNRNI | SAKAA | 69 | 98 | 120 | 84 | 171 | 188 | 163 | 104/92 | 82 | 170 |
|  | SVMNT | CNRNI | SGEAA | 81 | 98 | 120 | 84 | 168 | 188 | 163 | 101 | 82 | 173 |
|  | SVMNT | CNRNI | SGEAA | 81 | 98 | 123 | 87 | 165 | 188 | 163 | 101 | 82 | 176 |
|  | SVMNT | CNRNI | SAKAA | 66 | 98 | 117 | 84 | 171 | 188 | 163 | 107 | 85 | 170 |
|  | SVMNT | CNRNI | SGEAA | 81 | 98 | 120 | 84 | 168 | 188 | 163 | 101 | 85 | 173 |
|  | SVMNT | CNRNI | SGEAA | 69 | 95 | 132 | 84 | 168 | 188 | 163 | 101/98 | 85 | 185 |
|  | SVMNT | CNRNI | SGEAA | 81/69 | 101 | 132 | 75 | 168 | 188 | 163 | 101 | 82 | 185 |
|  | SVMNT | CNRNI | SGEAA | 78 | 98 | 132 | 90 | 171 | 188 | 163 | 113 | 82 | 185 |
|  | SVMNT | CNRNI | SAKAA | 66 | 98 | 132 | 84 | 171 | 188 | 163 | 107 | 82 | 185/170 |
|  | SVMNT | CNRNI | SGEAA | ND | 98 | 129 | 87/84 | 171/168 | 188 | 163 | 107 | 82 | 170 |
|  | SVMNT | CNRNI | SGKAA | 78 | 98 | 132/123 | 99 | 177 | 188 | 163 | 101 | 82 | 185 |
|  | SVMNT | CNRNI | CAKAA | 69 | 98 | 129 | 78 | 165 | 188 | 163 | 110 | 82 | 185/170 |
|  | SVMNT | CNRNI | SGEAA | 75 | 98 | 123 | 87 | 174 | 188 | 163 | 101 | 82 | 173 |
|  | SVMNT | CNRNI | SGEAA | 78 | 98 | 123 | 99 | 165 | 188 | 163 | 95 | 82 | 206 |
|  | SVMNT | CNRNI | SAKAA | 81/72 | 95 | 132/114 | 90 | ND | 191 | 172 | 104 | 82 | 185/176 |
|  | SVMNT | CNRNI | SGEAA | 93/90 | 98 | 129 | 87 | 171/168 | 188 | 163 | 107 | 82 | 170 |
|  | SVMNT | CNRNI | SGEAA | 66 | 98 | 132 | 84 | 168 | 188 | 163 | 107 | 82 | 185 |
|  | CVMNK | CNRNI | SAKAA | 69 | 98 | 132 | 87 | 168 | 188 | 163 | 107 | 82 | 170 |
|  | SVMNT | CNRNI | SAKAA | 69 | 98 | 120 | 84 | 171 | 188 | 163 | 107 | 82 | 170 |
|  | SVMNT | CNRNI | SAKAA | 75 | 98 | 123 | 87 | 168 | 188 | 163 | 110 | 82 | 173 |
|  | SVMNT | CNRNI | SAKAA | 69 | 95 | 120 | 90 | 174 | 188 | 163 | 104 | 82 | 170 |
|  | SVMNT | CNRNI | SGEAA | 81 | 98 | 132 | 87 | 165 | 188/191 | 163 | 113 | 82 | 176 |
|  | SVMNT | CNRNI | SAKAA | 75 | 98 | 123 | 84 | 168 | 188 | 163 | 107 | 85 | 173 |
|  | SVMNT | CNRNI | SAKAA | 69 | 95 | 120 | 87 | 174/171 | 188 | 163 | 107 | 82 | 170 |
|  | SVMNT | CNRNI | SGEAA | 81 | 101 | 132 | 87/75/93 | 165/168 | 188 | 163 | 104 | 82 | 185 |
|  | SVMNT | CNRNI | SAKAA | 72 | 98 | 132 | 93 | 165 | 206 | 199 | 107 | 82 | 170 |
|  | SVMNT | CNRNI | SGKAA | 72/81 | 95 | 132 | 84 | 168/171 | 188 | 163 | 98 | 82 | 173 |
|  | SVMNT | CNRNI | SGEAA | 75 | 98 | 132/117 | 78 | 168 | 188 | 163 | 107 | 82 | 173 |
|  | SVMNT | CNRNI | SAKAA + SAEAA | 75 | 95/98 | 132/117 | 84 | 168 | 188 | 163 | 98 | 82 | 170 |
|  | SVMNT | CNRNI | SGEAA | 69 | 98 | 120 | 87/99 | 186 | 191 | 163 | 98 | 82 | 170 |
|  | SVMNT | CNRNI | SAKAA | 72 | 95 | 132 | 99/87 | 171 | 188 | ND | ND | 82 | 173 |
|  | SVMNT | CNRNI | SAKAA | 69 | 95 | 132 | ND | 171 | 188 | 199 | 77 | 82 | 176 |
|  | SVMNT | CNRNI | SGEAA | 72 | 95 | 132 | 99 | 174 | 188 | 163 | 104 | 82 | 170 |
|  | SVMNT | CNRNI | SGEAA | 81 | ND | 120 | ND | ND | 188 | 199 | 77 | 88 | 194/185 |
|  | SVMNT | CNRNI | SGEAA | 78 | 98/95 | 120/132 | 99/96 | 165 | 188 | 163 | 98 | 82 | 170 |
|  | SVMNT | CNRNI | SGEAA | 75 | 98 | 132/117 | 84 | 168/165 | 188 | 163 | 101 | 82 | 173 |
|  | CVMNK | CNRNI | SGEAA | 84 | 101 | 126 | 87 | 171 | 188 | 163 | 101 | 85 | 206 |
|  | SVMNT | CNRNI | SAKAA | 69 | 95 | 120 | 87 | 174 | 188 | 163 | 107 | 82 | 173 |
|  | SVMNT | CNRNI | SGEAA | 69/66 | 98 | 144 | 98/95 | 168/171 | 188 | 163 | 98 | 82 | 179 |
|  | SVMNT | CNRNI | SAKAA | 81 | 95 | 132 | 84 | 171 | 245 | 163/169 | 104 | 82 | 173 |
|  | SVMNT | CNRNI | SGEAA | 93 | 95 | 141 | 87 | 171 | 188 | 163 | 107 | 82 | 176 |
|  | SVMNT | CNRNI | SAKAA | 69 | 98 | 132/117 | 84 | 171/168 | 188 | 163 | 107 | 82 | 170 |
|  | SVMNT | CNRNI | SGEAA | 69 | 98 | 129 | 87 | 171 | 188 | 163 | 107 | 82 | 179 |
|  | SVMNT | CNRNI | SAKAA | 69 | 95 | 129 | 90 | 168 | 188 | 163 | 107 | 82 | 170 |
|  | SVMNT | CNRNI | CAKAA | 72 | 98 | 132 | 87 | ND | ND | 163 | ND | 82 | 185 |
|  | SVMNT | CNCTI | SAKAA | 69 | 101 | 120 | 87 | 165 | 188 | 163 | 107 | 82 | 170 |
|  | SVMNT | CNRNI | SGEAA | 84 | 95 | 126 | 84 | 168 | 188 | 163 | 101 | 82 | 176 |
|  | SVMNT | CNRNI | SAKAA | 75/69 | 98 | 120/129 | 90/84 | 171/168 | 194 | 163 | 104 | 82 | 173/176 |
| 2011 | SVMNT | CNRNI | SGEAA | 81 | 98 | 120 | 84 | 168 | 188 | 163 | 101 | 82 | 173 |
|  | SVMNT | CNRNI | SGEAA | 81 | 98 | 120 | 84 | 168 | 188 | 163 | 101 | 82 | 170 |
|  | SVMNT | CNRNI | SAKAA | 69 | 95 | 126 | 87 | 171 | 188 | 163 | 107 | 82 | 173 |
|  | SVMNT | CNRNI | SGEAA | 81 | 98 | 120 | 84 | 150 | 188 | 163 | 101 | 82 | 152 |
|  | SVMNT | CNRNI | SGEAA | 81 | 98 | 120 | 87 | 165 | 188 | 163 | 113 | 85 | 176 |
|  | SVMNT | CNRNI | SGEAA | 69 | 95 | 129 | 87 | 165 | 188 | 163 | 104 | 82 | 164 |
|  | SVMNT | CNRNI | SGEAA | 69 | 101 | 129/132/120 | 75 | 165 | 188 | 163 | 101/98 | 82 | 194/191 |
|  | SVMNT | CNRNI | SGEAA | 81 | 98 | 123 | 87 | 168 | 188 | 163 | 101 | 85 | 173 |
|  | SVMNT | CNRNI | SGEAA | 81 | 95 | 132 | 96 | 147 | 245 | 163 | 104 | 82 | 197 |
|  | SVMNT | CNRNI | SGEAA | 69 | 95 | 123 | 87 | 168 | 188 | 163 | 101 | 82 | 206 |
|  | SVMNT | CNRNI | SGEAA | 93 | ND | 132 | 93 | ND | 188 | 163 | 107 | ND | 170 |
|  | SVMNT | CNRNI | SGEAA | 81 | 101 | 132/126 | 75 | 168 | 188 | 163 | 104 | ND | 206/185 |
|  | SVMNT | CNRNI | SGEAA | 72/81 | 95 | 132 | 87 | 174 | 188 | 163 | 107 | 82 | 173/169 |
|  | SVMNT | CNRNI | SGEAA | 81 | 98 | 120 | 84 | 168 | 188 | 163 | 101 | 82 | 173 |
|  | SVMNT | CNRNI | SGEAA | ND | 95 | 123 | 87 | 168 | 188 | 163 | 107 | 85 | 173 |
|  | SVMNT | CNRNI | SGEAA | 69 | 95 | 132 | 99 | 165 | 188 | 163 | 98 | 82 | 191 |
|  | SVMNT | CNRNI | SGEAA | 81 | 98 | 120 | 87 | 165 | 188 | 163 | 113 | 85 | 176 |
|  | SVMNT | CNRNI | SGEAA | 57/72 | ND | 132 | 87 | 174 | 188 | 163 | 107 | 76 | 173/185 |
|  | SVMNT | CNRNI | SGEAA | 81 | 101 | 123 | 87 | 165 | 188 | 163 | 107 | 85 | 206 |
|  | SVMNT | CNRNI | SGEAA | 72 | 95 | 132 | 84 | 174 | 188 | 163 | 107 | 82 | 173 |
|  | SVMNT | CNRNI | SGEAA | 60 | 98 | 129 | 87 | 168 | 188 | 169 | 107 | 82 | 164 |
|  | SVMNT | CNRNI | SGEAA | 81 | 98 | 120 | 87 | 165 | 188 | 163 | 113 | 85 | 176 |
|  | SVMNT | CNRNI | SAKAA | 84 | 95 | 120 | 84 | 180 | 188 | 163 | 107 | 82 | 164 |
|  | SVMNT | CNRNI | SGEAA | 72 | 98 | 132 | 84 | 177 | ND | 163 | 110 | ND | 185 |
|  | SVMNT | CNRNI | SGKAA + SGEAA | 69/75 | 98 | 126/120 | 75/93 | 168 | 188 | 163 | 98/107 | 82 | 176/173 |
|  | SVMNT | CNRNI | SGKAA | 69 | 98 | 126 | 93 | 168 | 188 | 163 | 98 | 82 | 176 |
|  | SVMNT | CNRNI | SGEAA | 69 | 101 | 126 | 87 | 171 | 188 | 163 | 107 | 85 | 206 |
|  | SVMNT | CNRNI | SGEAA | 81/78 | 98 | 132 | 93 | 147 | 188 | 163 | 98/95 | 82 | 161 |
|  | SVMNT | CNRNI | SAKAA | 81 | 98 | 132 | 81 | 183 | 188 | 163 | 113 | 79 | 170 |
|  | SVMNT | CNRNI | SGEAA | 81/78 | 95 | 132/123 | 87 | 165 | 188 | 163 | 107 | 85 | 173 |
|  | SVMNT | CNRNI | SGEAA | 69 | 101 | 126 | 87 | 171 | 188 | 163 | 107 | 82 | 206 |
|  | SVMNT | CNRNI | SAKAA | 69 | 95 | 132 | 87 | 171 | 188 | 163 | 104 | 82 | 173 |
|  | SVMNT | CNRNI | SGEAA | 69 | 98 | 129 | 87 | 171 | 188 | 163 | 107 | 82 | 179 |
|  | SVMNT | CNRNI | SGKAA | 57/69/84 | 95 | 132 | ND | ND | 188 | 163 | 104 | ND | 176 |
|  | SVMNT | CNRNI | SAKAA | 69 | 95 | 129 | 81 | 171 | 188 | 163 | 104 | 82 | 173 |
|  | SVMNT | CNRNI | SGEAA | 81 | 98 | 120 | 87 | 165 | 188 | 163 | 113 | 85 | 176 |
|  | SVMNT | CNRNI | SGEAA | 72 | 98 | 120 | 84 | 165 | 188 | 163 | 110 | 85 | 176 |
|  | SVMNT | CNRNI | SGEAA | 69 | ND | 132 | 99/90 | 186 | 248 | 163 | 98/101 | ND | 170/185 |
|  | SVMNT | CNRNI | SAKAA | 72 | 95 | 120 | 87 | 171 | 188 | 163 | 107 | 82 | 206 |
|  | SVMNT | CNRNI | SGEAA | 69/81 | 101 | 132/129/135 | 75/87 | 168 | 188/191 | 163 | 101/104 | 82 | 170 |
|  | SVMNT | CNRNI | SGEAA | 81 | 98 | 132 | 87 | 165 | 188 | 163 | 113 | 82 | 176 |
|  | SVMNT | CNRNI | SAKAA | 81 | 95 | 123 | 87 | 171 | 188 | 163 | 107 | 82 | 173 |
|  | SVMNT | CNRNI | SGEAA | 93 | 98 | 129 | 87 | 171 | 188 | 163 | 107 | 82 | 170 |
|  | SVMNT | CNRNI | SGEAA | 81 | 95 | 120 | 90 | 174 | 206 | 163 | 101 | 85 | 173 |
|  | SVMNT | CNRNI | SGEAA | 81 | 98 | 120 | 84 | 168 | 188 | 163 | 101 | 82 | 170 |
|  | SVMNT | CNRNI | SGEAA | 81 | 95 | 120 | 96 | 168 | 188 | 163 | 104 | 82 | 167 |
|  | SVMNT | CNRNI | SGEAA | 81 | 101 | 126 | 87 | 171 | 188 | 163 | 104 | 82 | 170 |
|  | SVMNT | CNRNI | SGEAA | 75 | 101 | 120 | 75 | 168 | 188 | 163 | 107 | 82 | 173 |
|  | SVMNT | CNRNI | SGEAA | 69 | 98 | 126/132 | 78 | 165 | 188 | 136 | 104 | 82 | 170 |
|  | SVMNT | CNRNI | SGEAA | 81 | 95 | 123 | 87 | 165 | 188 | 163 | 107 | 85 | 173 |
|  | SVMNT | CNRNI | SGEAA | 81 | 98 | 132/120 | 96 | 177 | 245 | 163 | 104 | 82/76 | 206 |
|  | SVMNT | CNRNI | SGEAA | 69 | 95 | 132 | 87 | 168 | 188 | 163 | 104 | 82 | 167 |
|  | SVMNT | CNRNI | SGEAA | 69 | 98 | 126/132 | 78 | 165 | 188 | 163 | 104 | 82 | 170 |
|  | SVMNT | CNRNI | SGEAA | 81 | 101 | 132 | 87/81 | 165 | 188 | 199 | 107 | 85/82 | 206 |
|  | SVMNT | CNRNI | SGEAA | 81 | 98 | 123 | 87 | 171 | 191 | 163 | 107 | 85 | 173 |
|  | SVMNT | CNRNI | ND | 57 | 95 | 132 | ND | ND | ND | 199 | 77 | 79 | 185 |
|  | SVMNT | CNRNI | SGEAA | 69 | 98 | 132 | 87 | 177 | 188 | 163 | 107 | 85 | 173 |
|  | SVMNT | CNRNI | SGEAA | 69 | 98 | 120/132 | 84 | 168 | 188 | 163 | 107 | 85 | 176 |
|  | SVMNT | CNRNI | SGEAA | 78 | 101 | 132 | 84 | 165 | 188 | 163 | 98 | 82 | 170 |
|  | SVMNT | CNRNI | SGEAA | 78/69/93 | 95 | 132/126 | ND | 165 | 188 | 163 | ND | 82 | ND |
|  | SVMNT | CNRNI | SAKAA | 72/69 | 98 | 123/120 | 99 | 168 | 188 | 163 | 110/98 | 82 | 164 |
|  | SVMNT | CNRNI | SGEAA | 69 | 95 | 120 | 81/99 | 177 | 245 | 166 | 107 | 82 | 173 |
|  | SVMNT | CNRNI | SGEAA | 69 | 101 | 117/132 | 90/84 | 165 | 191 | 163 | 101 | 85 | 170 |
|  | SVMNT | CNRNI | SGEAA | 75 | 101 | 120 | 75 | 168 | 188 | 163 | 107 | 82 | 173 |
|  | SVMNT | CNRNI | SGEAA | 69 | 95 | 129/132 | 87 | 165 | 188 | 163 | 104 | 82 | 164 |
|  | SVMNT | CNRNI | SGKAA + SGEAA | 75/72 | ND | 132 | 87/75 | 168 | 188 | 163 | 107 | ND | 173 |
|  | SVMNT | CNRNI | SGEAA | 72 | 98 | 129 | 84 | 168 | 188 | 163 | 104 | 82 | 179 |
|  | SVMNT | CNRNI | SGEAA | 69 | 98 | 132 | 93 | 168 | 191 | 163 | 107 | ND | 179 |
|  | SVMNT | CNRNI | SGEAA | 75 | 101 | 120 | 75 | 168 | 188 | 163 | 107 | 82 | 173 |
|  | SVMNT | CNRNI | SGEAA | 81 | 95 | 132 | 87 | 168 | 188 | 163 | 104 | 82 | 170 |
|  | SVMNT | CNRNI | SGEAA | 78 | 95 | 126 | 99 | 165 | 188 | 163 | 104 | 82 | 170 |
|  | SVMNT | CNRNI | SGEAA | 81 | 95 | 123 | 87 | 165 | 188 | 163 | 107 | 85 | 173 |

**Supplementary_FigS2. Pairwise comparisons of expected heterozygosity at each microsatellite locus between the three groups (2002/2003 vs. 2010 vs. 2011) and the permutation distributions under the null hypothesis.** The observed differences in expected heterozygosity are indicated by red arrows.


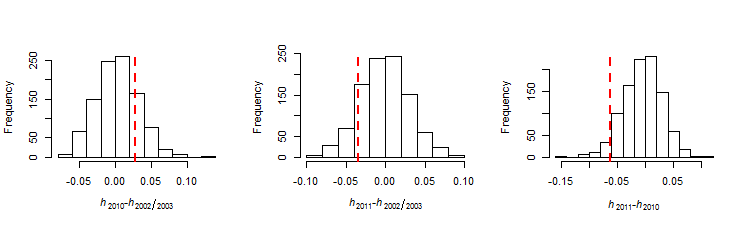


*P* = 0.408

*P* = 0.274

*P* = 0.102


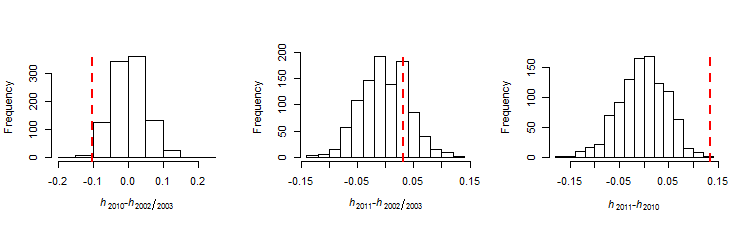


*P* = 0.010

*P* = 0.456

*P* = 0.000


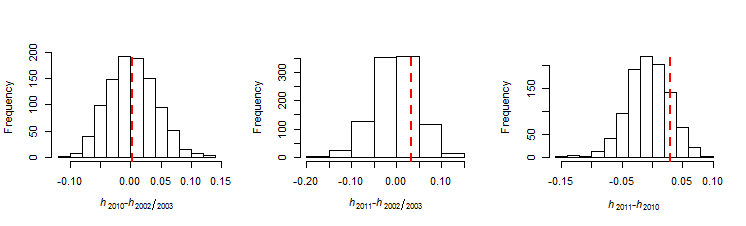


*P* = 0.986

*P* = 0.498

*P* = 0.306


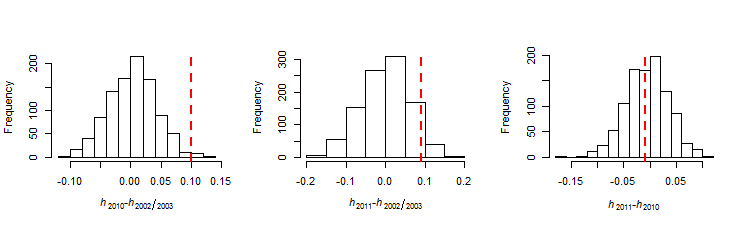


*P* = 0.018

*P* = 0.134

*P* = 0.946


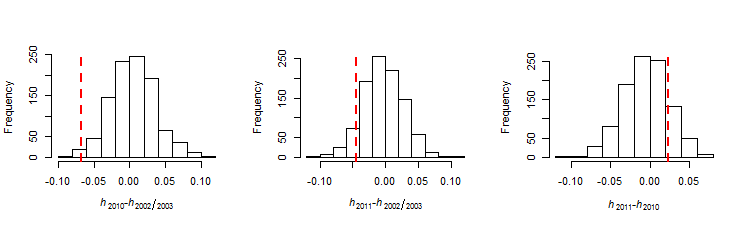


*P* = 0.014

*P* = 0.154

*P* = 0.318

ARA2

Pfg377

TA81

TA60

TA1


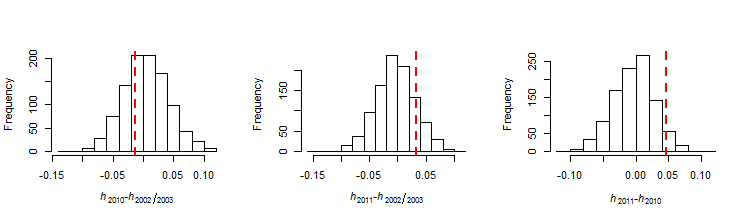

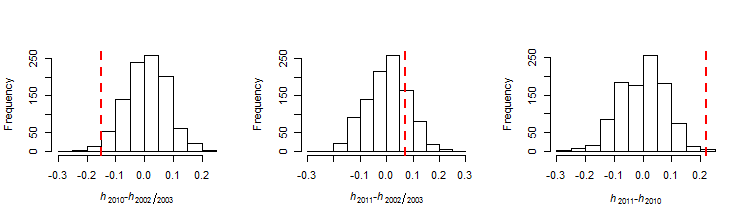

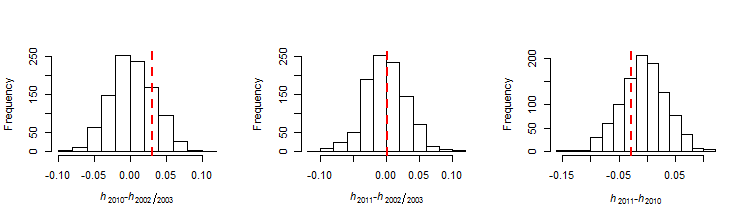

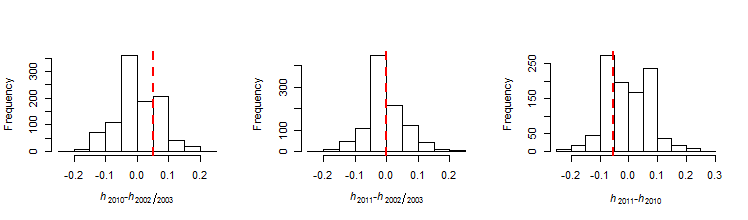

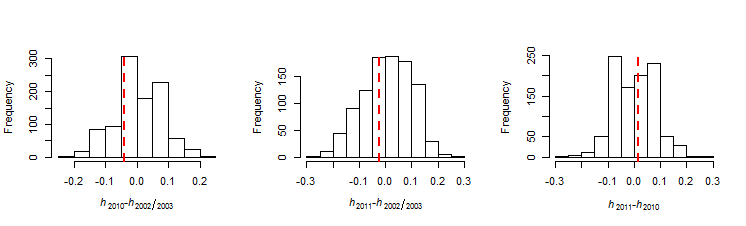


*P* = 0.662

*P* = 0.868

*P* = 0.810

*P* = 0.534

*P* = 0.824

*P* = 0.670

*P* = 0.386

*P* = 0.898

*P* = 0.558

*P* = 0.048

*P* = 0.384

*P* = 0.002

*P* = 0.638

*P* = 0.298

*P* = 0.100

TA42

TA109

TA87

2490

PfPK2

**Supplementary_FigS3.** **Principal component and Bayesian population structure analysis of single-clonal *Plasmodium falciparum* isolates.** (a) STRUCTURE was used to calculate the most probable number of clusters (K) in the sample set and then assign probabilities of membership to each cluster for each individual. The highest value of ΔK occurred at K = 3. Each individual is represented by a vertical bar displaying proportion of membership to each of clusters. (b) A total of 171 isolates with complete allele typing at 10 microsatellite markers were included in the analysis (46 in 2002/2003, 79 in 2010 and 46 in 2011). Each point represents an isolate. Proportions of variance are described in parenthesis under principal component (PC).


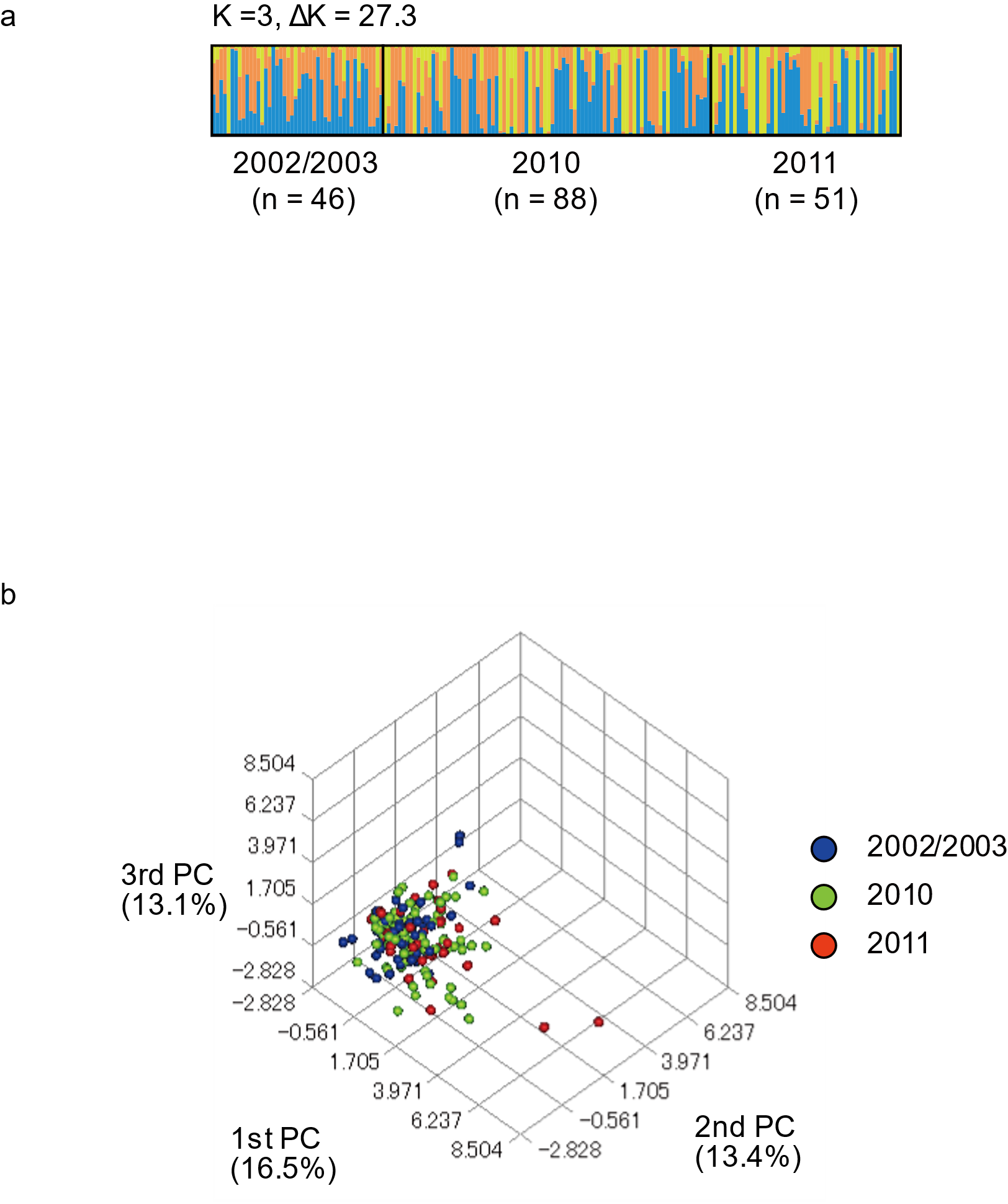

Supplement: Supplementary file 1 — Supplementary Information [file 41598_2018_23811_MOESM1_ESM.docx]
